# Nitrogen fertilization rates affect quality and curing characteristics of tobacco during the harvesting period under field chilling stress

**DOI:** 10.3389/fpls.2025.1681963

**Published:** 2025-10-29

**Authors:** Ke Ren, Zehui Wei, Kaiyuan Gu, Guorun Fu, Long Zhang, Hong Zhang, Bin Zhou, Feng Chen, Yi Chen, Khanom Simarani, Binbin Hu

**Affiliations:** ^1^ Yunnan Academy of Tobacco Agricultural Sciences, Kunming, Yunnan, China; ^2^ Division of Microbiology, Institute of Biological Sciences, Faculty of Science, Universiti Malaya, Kuala Lumpur, Malaysia; ^3^ College of Agronomy and Biotechnology, Engineering Research Center of South Upland Agriculture, Southwest University, Ministry of Education, Chongqing, China; ^4^ Chuxiong Tobacco Bureau of Yunnan Province, Chuxiong, China

**Keywords:** nitrogen fertilizer, curing characteristics, chilling stress, principal component analysis, physiological responses, *Nicotiana tabacum*

## Abstract

**Introduction:**

Field chilling stress during the maturation phase significantly impairs tobacco productivity and leaf quality. Nitrogen (N) management is a crucial agronomic approach for enhancing leaf quality and curing attributes; however, its specific role under chilling stress conditions remains poorly understood.

**Methods:**

Field demonstrations employed ‘Honghuadajinyuan’ tobacco cultivar under varying N fertilization rates, i.e., T1 (18.9 kg N ha^-1^), T2 (27 kg N ha^-1^, conventional rate), and T3 (35.1 kg N ha^-1^) with uniform basal application of 15,000 kg ha⁻¹ composted farmyard manure. This study evaluated the quality characteristics of fresh and cured tobacco leaves, as well as the curing process, by integrating physical and chemical analysis with multivariate statistical approaches, including principal component analysis and multiple linear stepwise regressions.

**Results:**

Fresh tobacco quality, such as leaf tissue integrity, chloroplast pigment content, and antioxidant enzyme activities as well as curing characteristics (leaf moisture regulation capacity, pigment conversion efficiency, and antioxidant system stability) exhibited gradient pattern of T3 > T2 > T1, respectively. This trend was also reflected in carbon-nitrogen metabolic accumulation, economic traits, and sensory quality of cured tobacco leaves. T3 treatment application enhanced tobacco yield (7.35%) and economic value (43.97%) as compared to T2 treatment. Principal component analysis and multiple linear stepwise regressions revealed covariance structures among economic traits, sensory quality, and principal components *F1* and *F2* (R^2^=0.87, *P*<0.05). *F1* (60.53% variance explanation rate) loaded predominantly on N fertilization rates and chloroplast pigments, whereas *F2* (23.75%) exhibited strong factor loading with nicotine content, total N, and neochlorogenic acid content.

**Conclusions:**

Increasing N fertilization by 30% above the conventional rate mitigates the adverse effects of field chilling stress, leading to significant improvements in yield and quality of mature tobacco.

## 1 Introduction

Nitrogen availability is a primary determinant of developmental progression, biomass accumulation (yield), and quality attributes in tobacco (*Nicotiana tabacum* L.) plants (Zhang et al., 2024a). During the growing season, effective management of N fertilizers can enhance crop tolerance efficiency, boost root vitality, upregulate photosynthetic leaf gas exchange, and support various physiobiochemical processes (Lyu et al., 2024). Crop plants manifested shortened stature, narrow and chlorotic leaves, reduced root activity, photosynthetic inhibition, antioxidant enzyme system disorder and dry matter accumulation/distribution in response to N deficiency (Ren et al., 2023b; Guan et al., 2025). Nitrogen deficiency limits plant reproductive development, reduces pollination and seed-setting rates, and downregulated yield and quality, while sufficient N fertilization prevents these impacts (Parry et al., 2025). Excessive N fertilization impaired plant growth, reproductive development, soil fertility and yield, and also improved pest and disease tolerance capacity, environmental pollution, and cultivation costs (Gelaye, 2024). Low and excess N synergistically impair tobacco leaf development and accumulation of chemical components, particularly nicotine biosynthesis, which is critical for tobacco leaf quality (Liu et al., 2022b). Suboptimal N availability limits root functionality and alkaloid synthesis, whereas optimal N management enhance leaf quality (Zhu et al., 2024). Additionally, excess fertilization should be avoided due to reduced N utilization efficiency and delayed maturation (Feng et al., 2024b).

Chilling stress (0–20°C) disrupts the photosynthetic apparatus, membrane integrity, and C/N metabolic fluxes in plants, leading to yield reduction through impaired organogenesis and chloroplast dysfunction (Goswami et al., 2022; Zhang et al., 2024b). Chilling stress induces histomorphological abnormalities, including chlorosis, tissue dehydration, and premature senescence that compromise curing characteristics and leaf quality of tobacco plants (Li et al., 2021). Strategic N supplementation has emerged as a critical mitigation strategy, enhancing cold acclimation by stabilizing chloroplast pigments, optimizing photosynthetic quantum efficiency of photosystem II and antioxidant system (Govindasamy et al., 2023; Qi et al., 2024). This approach is particularly effective in Yunnan’s high-altitude tobacco systems (>2000 m), where chilling stress prevalence enhances with elevation (He et al., 2020). N-mediated regulation of moisture and physiobiochemical processes demonstrates unique efficacy in maintaining membrane lipid homeostasis and metabolic equilibrium under low-temperature conditions. Current research gaps persist regarding N regulation impacts on *Nicotiana tabacum* quality in Yunnan’s altitudinal agroecosystems, primarily constrained by methodological complexities in extreme environments. Elucidating the molecular-physiological nexus of tobacco adaptation under chilling stress is pivotal for developing precision N management frameworks that synchronize fertilizer efficiency with chilling stress resilience, a critical pathway to mitigate field-scale membrane lipid peroxidation and photosynthetic apparatus degradation.

As a thermophilic crop, tobacco exhibits impaired quality and curing characteristics under low-temperature conditions through disruptions to leaf structure, photosynthesis, C/N metabolism, and plasma membrane systems (Gu et al., 2024). Increased N fertilization rates mitigates low-temperature impacts by promoting tobacco leaf tissue development, enhancing photosynthetic capacity and functionality of the leaf plasma membrane systems (Chen et al., 2025). Although increasing the N fertilization levels induces hypertrophy in mature leaves and delays maturation process (Qiang et al., 2023). This provides a crucial prerequisite for the yield of flue-cured tobacco in subsequent stages, rather than completely diminishing the value of flue-cured tobacco. Elevated N fertilization rates modulates the efficiency of enzymatic kinetics within the C-N metabolic flux, particularly affecting the activities of sucrose synthase, sucrose phosphate synthase, and nitrate reductase activities (Sun et al., 2023). Enhanced N fertilization rates downregulated amylase activity during maturation stage while delaying the shift from N to C metabolism, leading to reduced starch accumulation and elevated protein, nicotine, and nous compounds (Ma et al., 2025). Additionally, increased N fertilization rates results in excessive N metabolism in plants at maturity stage, which in turn inhibits the metabolism and synthesis of polyphenols (Wang et al., 2021). The primary way of chilling stress harming plants by damaging the plasma membrane and inhibiting the activity of protective enzymes (Gu et al., 2024). Inadequate N supply under chilling stress exacerbates chilling damage by inhibiting plant development and reducing stress resistance capacity (Ban et al., 2025). Sufficient N fertilizer supply can significantly enhance the enzymatic activity of plants, thereby improving its tolerance (Qi et al., 2023).

Nitrogen fertilization rates directly influence the growth and yield of plants, as a key agronomic management factor, with optimized application enhancing economic benefits (Zhang et al., 2023a). There have been a lot of research findings on the effects of different N fertilization rates and chilling stress on tobacco plants, but most of the relevant studies are limited to a single factor, and different fertility and natural conditions. Chilling stress synergistically affects tobacco during the harvesting period have rarely been reported. Currently, numerous studies have assessed the impact of N fertilization rate on growth and development of tobacco plants (Zhu et al., 2024; Khan et al., 2025). The combined effects of chilling stress under varying fertility and natural conditions during the harvest period have yet to be reported. The response mechanism of tobacco to low-temperature stress under natural environmental conditions under different N fertilization rates urgently needs to be studied. This study investigated tobacco production systems in high-altitude ecoregion of Yunnan Province, China where chronic chilling stress fundamentally constrains crop quality. By combining principal component analysis (PCA) with multiple linear stepwise regressions (MLSR), we established a robust analytical framework to interpret multidimensional quality parameters and identify key parameters of tobacco quality and curing characteristics. This methodology provides novel insights for optimizing postharvest processing and enhancing chilling resilience in field-grown tobacco. This study aims to explore the correlation between N fertilization regimes and tobacco quality attributes under field chilling stress. The primary objectives focus on optimizing N fertilization rates under field chilling stress, improving leaf curing characteristics and economic traits, resolving challenges associated with altitudinal cultivation shifts, and safeguarding the economic viability of production. These were beneficial to in-depth study of the relationship among the factors affecting the quality characteristics of cured tobacco leaves under chilling stress. Therefore, this study based on the field demonstration under chilling conditions in Yunnan province, China high-altitude (Above 2500 m) agroecosystem (maximum temperature difference within a month exceeds 25°C) (He et al., 2020). It is great significance to explore the relation between various N fertilizer rates and the damage caused by chilling stress on tobacco plants.

## 2 Materials and methods

### 2.1 Experimental location and design

**Figure 1:**
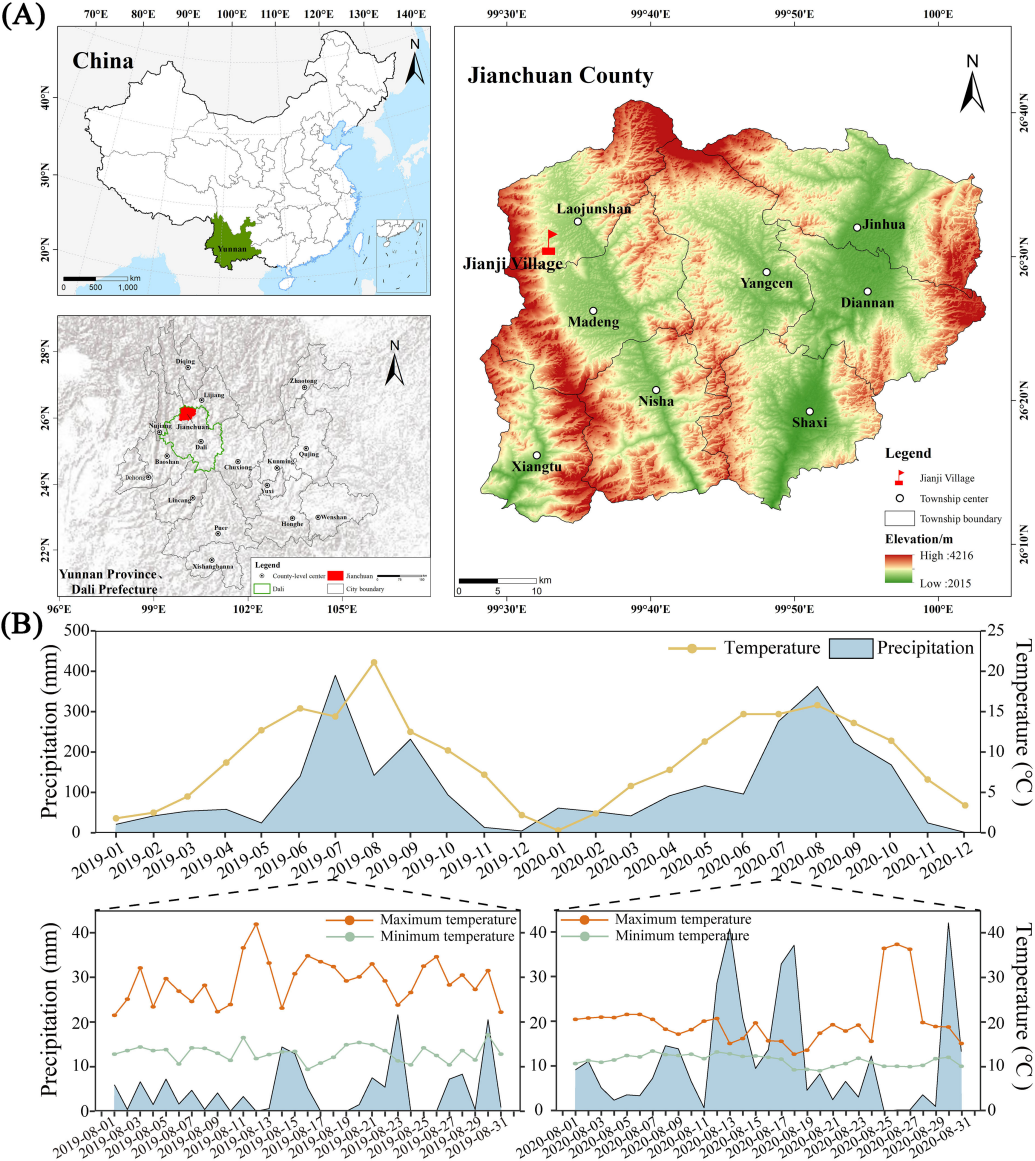
Study region, station locations, and climatic observations. Map showing the location of the experimental site **(A)**. The monthly average temperature, average rainfall, and temperature and rainfall in August at the test site were measured using the WH-2310 wireless weather station (Jiaxing Misu Electronic Co., Ltd.) from 2019 to 2020 **(B)**. The x-axis of figure B represents the experiment duration.

The experiment was conducted in Laojun Mountain Town (26°31’N, 99°33’E; 2565 m altitude) characterized by a continental plateau monsoon climate, with mean annual temperature of 9°C and precipitation of 113.9 mm (2019-2020). Maximum diurnal thermal amplitude reached 30.1°C (2019) and 27.3°C (2020) during August phenophases (**Figure 1**). The field sandy red soil (silty loam) classified as Ultisol (USDA, 2014) from mid-altitude tobacco-growing zones (1800–2600 m). It has pH 6.47 and contains soil organic matter (56.19 g kg^−1^), total N (2.76 g kg^−1^), total phosphorus (1.11 g kg^−1^), total potassium (17.64 g kg^−1^), soluble N (210.8 mg kg^−1^), available phosphorus (91.3 mg kg^−1^), and available potassium (285.5 mg kg^−1^) at 0–15 cm of soil depth from the surface.

The locally adapted flue-cured tobacco cultivar ‘Honghuadajinyuan’ (HD) was selected for research demonstration resilience to chilling stress in the mountainous ecoregions of Yunnan, China. The young seedlings bred under membranes were transplanted on 13–15 April, with the row and plant spacing being 120 × 60 cm. The seedlings were topped from 10–13 June, during which 15 or 16 leaves were left. Lower leaves were picked during 3 to 4 July and curing completed on 7 to 11 September. The experiment set as a randomized block design (RBD), 3 treatments with different N fertilization rates were set, and each treatment test plot of 12 m long and 8 m wide. The experiment employed as a randomized complete block design (RCBD) with three N fertilization rates, such as T1 (18.9 kg N ha^-^¹), T2 (27 kg N ha^-^¹, conventional rate), and T3 (35.1 kg N ha^-^¹), each supplemented with 15000 kg ha^-^¹ composted farmyard manure as basal application. Fertilization protocols were implemented according to local agronomic practices. Phosphorus fertilizer (80 kg P_2_O_5_ ha^-^¹) and potassium sulfate (300 kg ha^-^¹; K_2_O ≥52%, Cl^-^ ≤1.0%, S ≥17.5%) were applied. A tobacco-specific compound fertilizer (N: P_2_O_5_: K_2_O=12:10:25) served as basal application, supplemented with 15000 kg ha^-^¹ composted farmyard manure. Top-dressing was applied using a 16:0:25 NPK formulations at 15 and 30 days after transplantation.

Middle-positioned leaves (8–12^th^ phyllotactic nodes) were harvested during standard maturation windows and uniformly arranged on curing rods. The leaves were sorted by visual quality and loaded at an optimal density (100–120 leaves/rod) in regionally standardized bulk-curing barns. A three-tiered curing system (150–170 rods/tier) implemented protocols detailed in **Supplementary Table S1**.

### 2.2 Observation of anatomical structures

The microstructures of fresh tobacco leaves before harvesting and curing were observed according to the following method, i.e., nearly about 0.5 × 0.5 cm leaves were cut from between the right leaf tip and the 6 to 7^th^ branch veins of fresh tobacco leaf samples. The samples were fixed in FAA (50%, v/v, alcohol), processed using a conventional paraffin sectioning method, with a slice thickness of 10 μm. Hematoxylin staining applied and the sections were sealed with Canadian gum to make permanent for observation and analysis by Olympas microscope. A total of 2 sections were examined with 5 visual fields observed in each section. The average measurements were calculated for each section. The leaf thickness (LT) as well as the thickness of the upper epidermis (UE) thickness, lower epidermis (LE), palisade tissue (PT) and spongy tissue (ST) were observed, and data statistically analyzed.

### 2.3 Determination of tobacco leaf water loss efficiency

The fresh tobacco leaves were dried to remove surface moisture and the samples in each stage of the curing process were weighed by electronic balance (American Shuangjie SA-200Y). The weights were recorded as FW_0_ and FW_n_ (38, 42, 48 and 54°C), respectively. Then the fresh tobacco leaves and the samples of each stage in the curing stages were repeatedly put into the blast oven-dried at 105°C for 1 h (initial deactivation) followed by 60°C for 48 h (upto constant weight). The samples were weighed again and recorded as DW_0_ and DWn (38, 42, 48 and 54°C), respectively.


(1) $$\text{Moisture content of fresh tobacco leaves}(\%)=[(\text{F}{\text{W}}_{0}-\text{D}{\text{W}}_{0})/\text{F}{\text{W}}_{0}]\times 100\%$$

(2) Moisture content of tobacco leaves at different curing stages(%)=[(FWn−DWn)/FWn]×100%

(3) $$\text{Water loss rate of tobacco leaves}(\%)=[(\text{F}{\text{W}}_{0}-\text{FWn})/(\text{FW}0-\text{DW}0)]\times 100\%$$

In these Equations 1–3, the fresh weight at the initial state (FW_0_) represents the pre-curing biomass. Dry weight baseline (DW_0_) was determined by heat fixation at 105°C for 1 hr, followed by oven drying at 60°C until a constant weight was achieved (less than 0.5% variation between consecutive measurements). Stage-specific fresh (FW_n_) and dry weight (DW_n_) were measured at four curing temperatures (38, 42, 48, and 54°C), with DW_n_ samples subjected to the same thermal stabilization and drying protocols as DW_0_.

### 2.4 Determination of physiochemistry and chloroplast pigment indexes

The measurement of antioxidant enzymes were immediately taken after sampling in each stage. The sample was put into liquid N, and then transferred to the refrigerator at -80°C to determine for biochemical analysis, including superoxide dismutase (SOD), peroxidase (POD), catalase (CAT), malondialdehyde (MDA) and polyphenol oxidase (PPO). The activity of each enzyme was determined by the kit produced by Suzhou Keming Biotechnology Co., Ltd., Suzhou, China, as per instructions.

Measurement of chemical indices, such as starch, total sugar, reducing sugar, total N and nicotine of tobacco samples in different stages were mainly detected using the following methods. The contents of the total sugar and reducing sugar were analyzed according to Tobacco and Tobacco Products–Determination of Water Soluble Sugars–Continuous Flow Method (YC/T159-2002). Total N was analyzed based on the Tobacco and Tobacco Products–Determination of total N–continuous Flow Method (YC/T161-2002). Nicotine was analyzed by utilizing Tobacco and Tobacco Products–Determination of Nicotine–Continuous Flow Method (YC/T160-2002). The content of starches was measured by referring to Tobacco and Tobacco Products–Determination of Starch–Continuous Flow Method (YC/T216-2014). The amounts of chlorogenic acid, scopoletin, rutin, neochlorogenic acid, caffeic acid, lutein and β-carotene were mainly measured using the method recommended in YC/T 202–2006. Photosynthetic pigments (Chl. a, Chl. b & Chl. a+b) and carotenoids were determined by an ethanol-spectrophotometer in tobacco leaves at different stages.

### 2.5 Determination of economic traits and sensory quality of cured tobacco leaves

Economic traits were quantified according to the GB2635–92 grading standards by local tobacco stations. Market-aligned pricing facilitated the calculation of premium-grade proportions and average values. Sensory attributes were evaluated by seven experts from the Yunnan Tobacco Technology Center using provincial standards. They assessed style characteristics (aroma notes), aroma attributes (volume and quality), sensory properties (concentration, off-odors, strength, irritation), and taste profiles (flavor, cleanliness, moisture) on 100-point scale.

### 2.6 Data analysis

The data were analyzed by Microsoft Excel 2016 and SPSS version 26.0 statistical software. Correlation analysis, PCA and MLSR were used to establish a model based on different indexes of fresh tobacco quality, curing characteristics and key chemical components, etc., to comprehensively evaluate the effects of different N fertilization rates on the yield and quality of tobacco under field chilling stress. The specific data processing steps are divided into Pearson correlation analysis and MLSR (**Appendix A, Supplementary data**). For the rest of the data, we used repeated measures analyses of one-way repeated measures in SPSS 26.0 (IBM SPSS Statistics Inc., Chicago, IL, USA) to examine the effects of different N fertilization rates on leaf tissue structure, water loss capacity and plastid pigment of tobacco, five of regular chemical component, polyphenols, antioxidant enzyme activity index. Shapiro-Wilk normal distribution test and Mauchly’s test of sphericity were conducted on each data. When *P*>0.05 was considered to meet the spherical hypothesis test, if not, the correction coefficient Epsilon> of Greenhouse-Geisser was compared at 0.75. Huynh-Feldt degree of freedom correction was used, otherwise, Greenhouse-Geisser method was used for degree of freedom correction (Haverkamp and Beauducel, 2017). One-way analysis of variance was used to determine the effects of N fertilization rates on tissue structure, yield and quality of fresh tobacco leaves and sensory assessment. Values used for statistical analysis represent the average of three biological replicates with standard error. A *post hoc* test (Bonferroni, *P* < 0.05) was used for determining the significance levels of different studied parameters. The chart was designed with Sigma Plot 14.0 (Systat Software, Inc. USA).

## 3 Results

### 3.1 Effects of nitrogen regimes on chilling-induced anatomical dynamics

**Figure 2:**
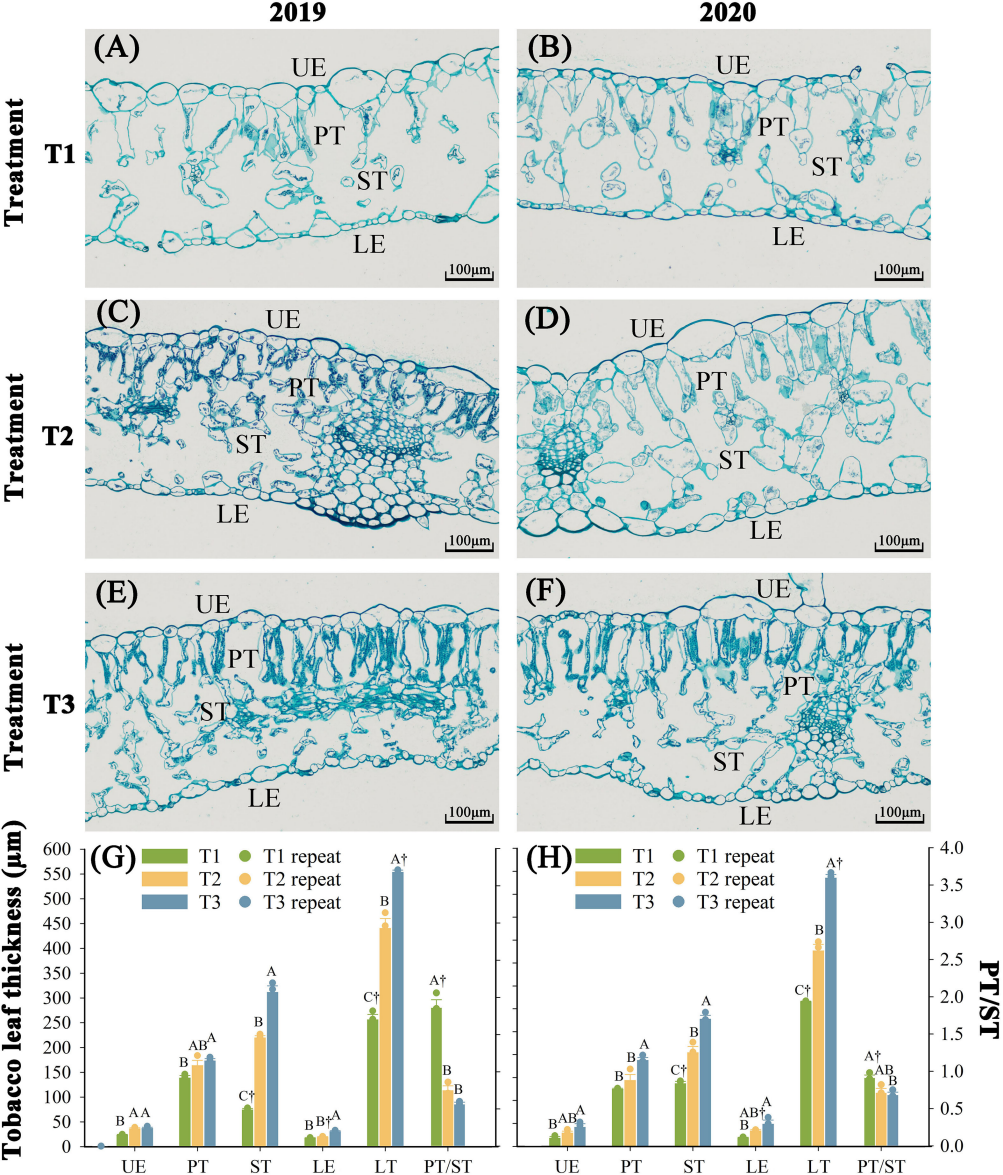
Effect of different N fertilization rates on tissue structure of fresh tobacco leaves. **(A)** The tissue structure of fresh tobacco leaves of T1 treatment in 2019. **(B)** The tissue structure of fresh tobacco leaves of T1 treatment in 2020. **(C)** The tissue structure of fresh tobacco leaves of T2 treatment in 2019. **(D)** The tissue structure of fresh tobacco leaves of T2 treatment in 2020. **(E)** The tissue structure of fresh tobacco leaves of T3 treatment in 2019. **(F)** The tissue structure of fresh tobacco leaves of T3 treatment in 2020. **(G)** The tissue structure thickness of fresh tobacco leaves in 2019. **(H)** The tissue structure thickness of fresh tobacco leaves in 2020. UE, upper epidermis; PT, palisade tissue; ST, sponge tissue; LE, lower epidermis; LT, leaf thickness; PT/ST, palisade tissue/sponge tissue. The values of PT/ST are controlled by the right ordinate, and other indexes are controlled by the left ordinate. T1, N 18.9 kg·ha^-1^ + Rot farm manure 15000 kg·ha^-1^; T2, N 27 kg·ha^-1^ + Rot farm manure 15000 kg·ha^-1^, which is the normal local N fertilizer rate; T3, N 35.1 kg·ha^-1^ + Rot farm manure 15000 kg·ha^-1^. Different letters indicate significant differences between different treatments in the same year (*P* < 0.05). “†” indicates significant differences between the same treatments in different years (*P* < 0.05). The X-axis in figures **(G, H)** represents the indicators of the tobacco leaf tissue structure.

Nitrogen fertilization regimes differentially regulated foliar cellular architecture with significant structural alterations (*P* < 0.05) observed over two consecutive years (**Figure 2**). T1 (18.9 kg N ha^-^¹) exhibited compromised epidermal integrity, with ruptured lower epidermis (LE), deformed palisade (PT) and spongy tissues (ST), expanded intercellular spaces, and reduced leaf thickness (LT). T2 (27 kg N ha^-^¹) maintained intact LE but showed partial PT/ST disorganization and constricted intercellular pores. In contrast, T3 (35.1 kg N ha^-^¹) demonstrated optimal cellular architecture, tightly packed epidermal cells, well-developed PT/ST with sediment deposition, and maximized LT. Over two consecutive years, tissue thickness followed T3 > T2 > T1 (*P* < 0.05). Compared to T2, T3 increased upper epidermis (UE, 22.3%), PT (17.9%), ST (38.7%), LE (49.6%), and LT thickness (31.4%), respectively. Relative to T1, these increments reached 35.9% (UE), 14.3% (PT), 49.4% (ST), 26.4% (LE), and 33.9% (LT). Notably, T3 reduced PT/ST structural anomalies by 88.9% and 14.9% compared to T1 and T2, respectively.

The results of the Kaiser-Meyer-Olkin (KMO) test (≥0.5) and Bartlett’s sphericity test (*P* < 0.05) indicate a strong correlation among the variables, satisfying the assumptions required for principal component analysis (PCA) (**Supplementary Table S2**). The first principal component, extracted according to standard eigenvalue criteria, exhibited initial eigenvalues ≥ 4.95 and accounted for at least 82.27% of the cumulative contribution rate across both experimental years. Significant treatment effects (*P* < 0.05) on leaf tissue structural indices were observed between two years. Compared to T2, T3 showed a 3690.5% increase in composite structural scores, whereas T1 demonstrated 3691.7% reduction.

### 3.2 Effects of nitrogen regimes on chilling-induced water loss dynamics in tobacco leaves during curing stages

**Figure 3:**
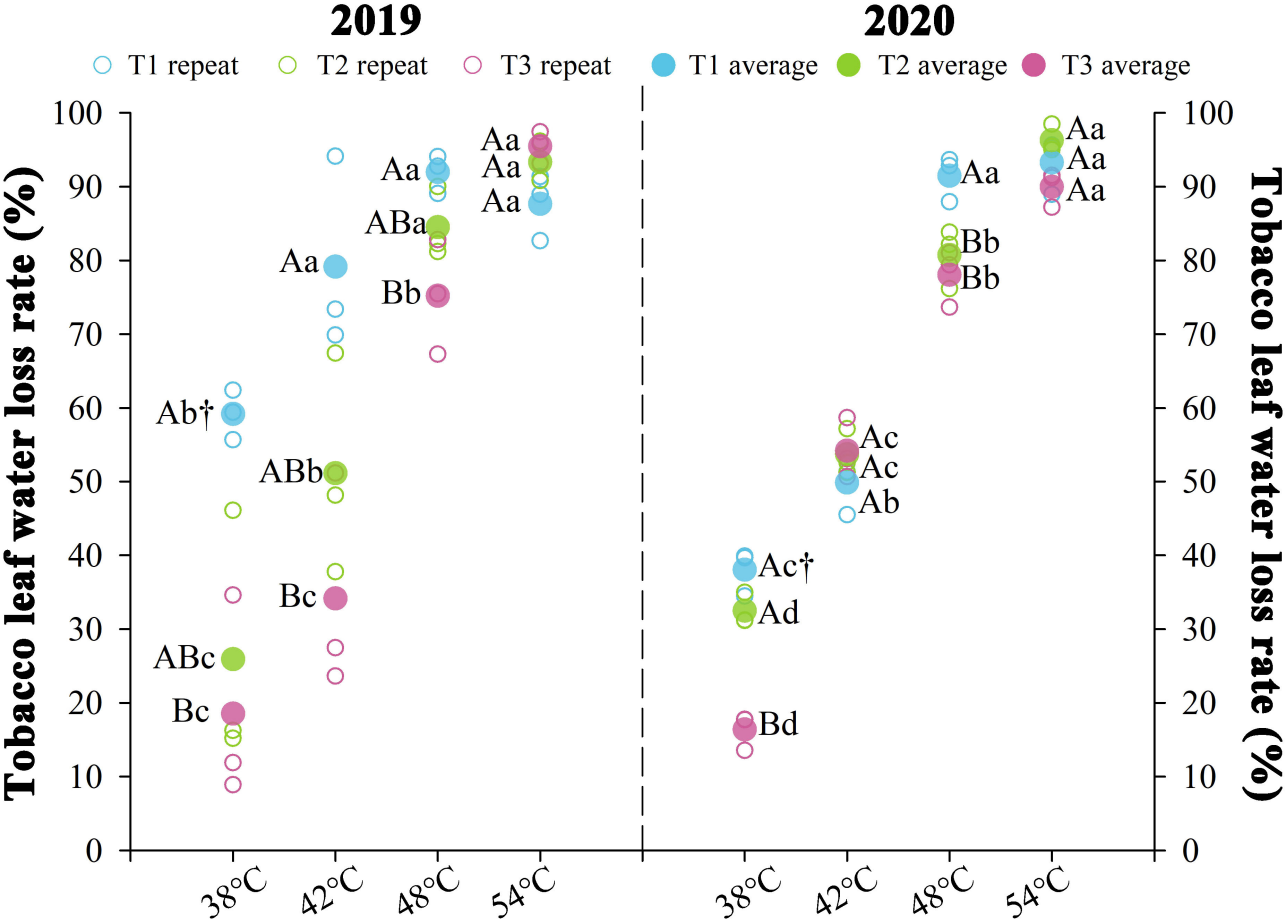
Effect of different N fertilization rates on water loss rate of tobacco leaves during curing stages. Different capital letters indicated that there were significant differences between the same year, the same curing stage and different treatments (*P* < 0.05). Different lowercase letters indicated significant difference between the same year, different curing stages and same treatment (*P* < 0.05). “†” indicates significant differences between different years, curing stage, and treatments (*P* < 0.05).

Nitrogen fertilization regimes differentially modulated leaf water loss rates, with significant changes in water loss (*P* < 0.05) observed over two consecutive years during curing stages (**Figure 3**). The results indicated that the moisture content of tobacco leaves underwent a rapid water loss phase between 38 and 48°C, followed by a slower water loss phase from 48 to 54°C as the temperature enhanced. The water loss rates for each treatment decreased in T1 > T2 > T3. Significant differences were primarily observed during the rapid water loss phase of the curing process. At the same temperature point in this phase, the average water loss rate of T3 treatment decreased by 20.84% compared to T2, while the average water loss rate of T1 increased by 35.99%. The average water loss rates during the rapid water loss stage for T1 were 0.72%·h^-^¹ (38°C), 0.69%·h^-^¹ at (42°C), and 0.76%·h^-^¹ (48°C), respectively. T2 exhibited average water loss rates of 0.43%·h^-^¹ (38°C), 0.56%·h^-^¹ (42°C), and 0.68%·h^-^¹ (48°C). Under identical thermal conditions, T3 exhibited average water loss rates of 0.26, 0.47, and 0.63%·h^-^¹, respectively. During the rapid dehydration phase, T1 exhibited water loss rates of 0.72% h^-^¹ (38°C), 0.69% h^-^¹ (42°C), and 0.76% h^-^¹ (48°C). T2 exhibited rates of 0.43, 0.56, and 0.68% h^-^¹ at corresponding temperatures, while T3 demonstrated enhanced hydraulic stability with 0.26, 0.47, and 0.63% h^-^¹ under the same thermal conditions.

### 3.3 Influence of nitrogen regimes on chilling-induced plastid pigment dynamics in tobacco leaves during curing stages

**Figure 4:**
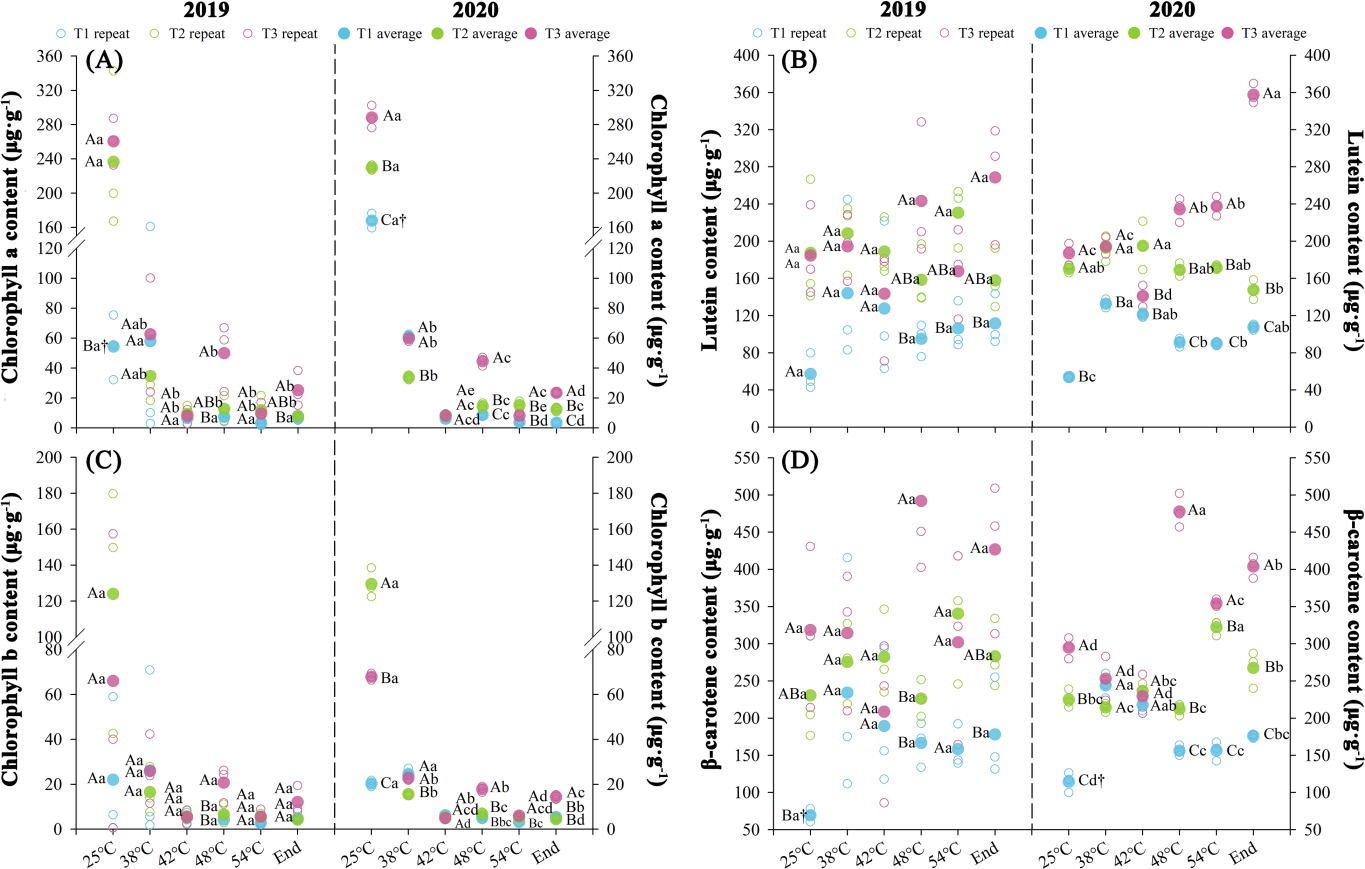
Effect of different N fertilization rates on photosynthetic pigment, such as chlorophyll a **(A)**, lutein content **(B)**, chlorophyll b **(C)**, and β-carotene content **(D)** of tobacco during curing stages at different ambient air temperature. Different capital letters indicated that there were significant differences between the same year, the same curing stage and different treatments (P < 0.05). Different lowercase letters indicated significant difference between the same year, different curing stages and same treatment (P < 0.05). “†” indicates significant differences between different years, curing stage, and treatments (P < 0.05).

Nitrogen fertilization regimes differentially regulated photosynthetic pigment profiles, with significant variations (*P* < 0.05) observed across consecutive over two consecutive years during curing stages (**Figure 4**). The results showed that the overall photosynthetic pigment contents in each treatment followed the order T3 > T2 > T1. Chlorophyll a and b gradually downregulated throughout the curing stages, while the contents of lutein and β-carotene initially decreased and then increased. Over two consecutive years, throughout the curing stages, T3 exhibited higher phytopigment concentrations than T2, with increase of chl a (76.98%), chl b (67.59%), lutein (22.20%), and β-carotene (34.62%). In contrast, T1 showed average reductions of 27.29% (chl a), 12.68% (chl b), 42.88% (lutein), and 32.50% (β-carotene) relative to T2 across both demonstration years. The contents of chl a and chl b significantly decreased at temperatures ranging from 25 to 42°C (*P* < 0.05). The average degradation rates of chl a in each treatment were 1.12 μg·g^-^¹·h^-^¹ (T1), 2.41 μg·g^-^¹·h^-^¹ (T2), and 2.68 μg·g^-^¹·h^-^¹ (T3). For chl b, the average reduction rates in each treatment were 0.17 μg·g^-^¹·h^-^¹ (T1), 1.30 μg·g^-^¹·h^-^¹ (T2), and 0.66 μg·g^-^¹·h^-^¹ (T3), respectively.

The results of the KMO test (≥0.5) and Bartlett’s sphericity test (*P* < 0.05) indicate a strong correlation among the variables, satisfying the assumptions required for PCA (**Supplementary Table S3**). The first principal component, extracted according to standard eigenvalue criteria, exhibited initial eigenvalues ≥ 1.39 and accounted for at least 91.36% of the cumulative contribution rate of both experimental years. Significant differences (*P* < 0.05) in composite photosynthetic pigment scores were observed across curing stages in both experimental years. Compared to T2, T3 exhibited an average increase of 429.27% in composite scores, whereas T1 showed an average reduction of 371.88%.

### 3.4 Effects of nitrogen regimes on chilling-induced chemical composition dynamics during curing stages

**Figure 5:**
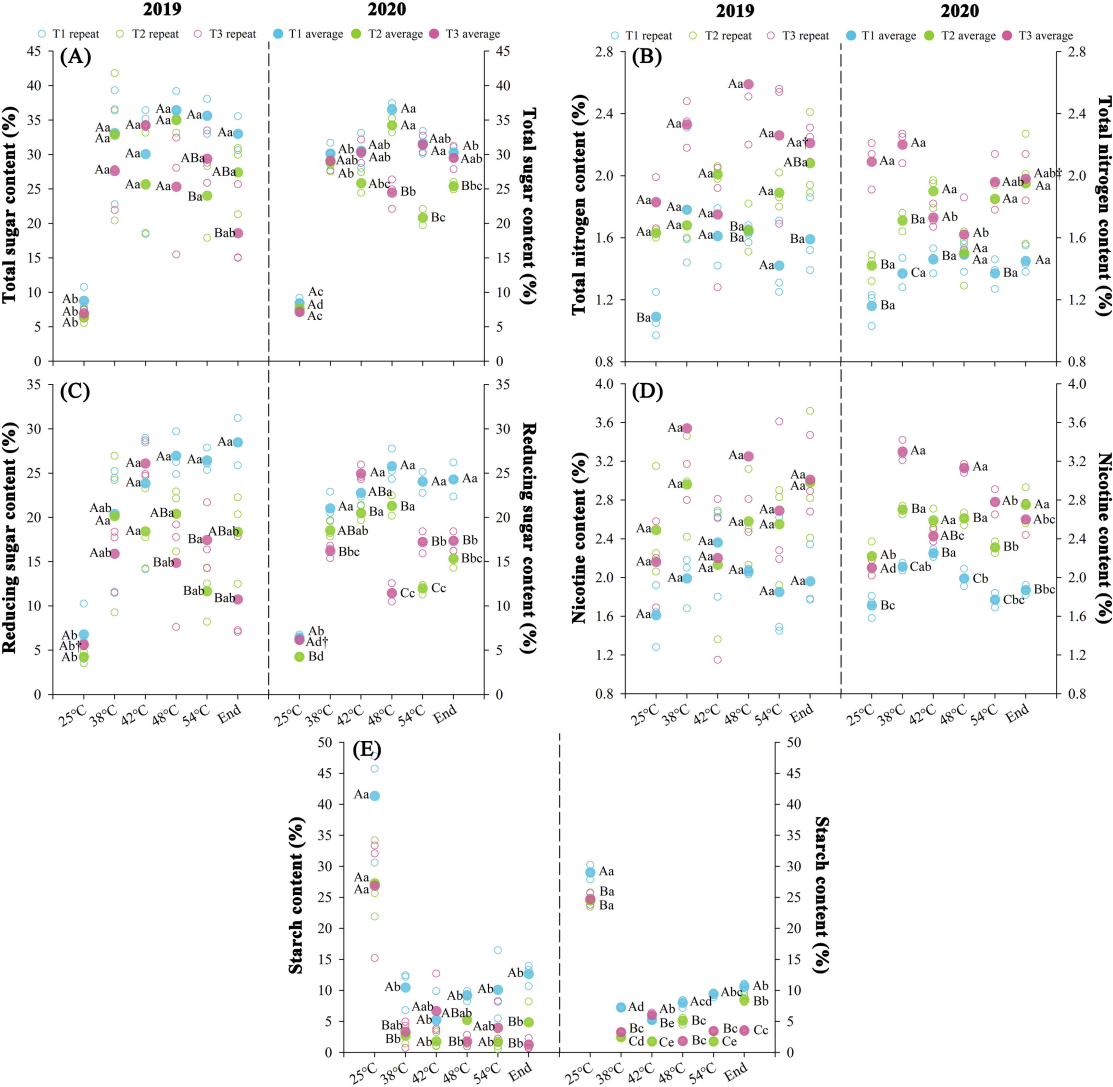
Effect of different N fertilization rates on total sugar **(A)**, total N **(B)**, reducing sugar **(C)**, nicotine **(D)**, and starch content **(E)** of tobacco during curing stages at different ambient air temperature. Different capital letters indicated that there were significant differences between the same year, the same curing stage and different treatments (P < 0.05). Different lowercase letters indicated significant difference between the same year, different curing stages and same treatment (P < 0.05). “†” indicates significant differences between different years, curing stage, and treatments (P < 0.05).

Nitrogen fertilization regimes differentially modulated chemical composition profiles with significant variations (*P* < 0.05) observed in curing stages of both years (**Figure 5**). T1 demonstrated superior total sugar, reducing sugar, and starch accumulation (*P* < 0.05), while total N and nicotine contents followed T3 > T2 > T1. During thermal progression through yellowing stage (38°C) to stem drying stage (54°C), soluble sugars exhibited biphasic accumulation patterns (rapid/sustained phases), contrasting with starch’s inverse biphasic degradation trajectory. Over two consecutive years, throughout the curing stages, T1 exhibited elevated carbohydrate accumulation, total sugar (+19.7%), reducing sugar (+46.8%), and starch (+182.1%) compared to T2 treatment, alongside decreased total N (-17.5%) and nicotine (-23.0%). Conversely, T3 showed modest increases in total N (+16.9%) and nicotine (+7.3%) relative to T2, while carbohydrate levels remained statistically unchanged. At temperatures ranging from 25 to 42°C, total sugar and reducing sugar contents increased significantly (*P* < 0.05), while starch content reduced significantly (*P* < 0.05). The average synthesis rates of total sugar were 0.23%·h^-^¹ (T1), 0.20%·h^-^¹ (T2), and 0.27%·h^-^¹ (T3), reducing sugars showed synthesis rates of 0.18%·h^-^¹ (T1), 0.16%·h^-^¹ (T2), and 0.21%·h^-^¹ (T3), with concurrent starch degradation rates of 0.32%·h^-^¹ (T1), 0.26%·h^-^¹ (T2), and 0.21%·h^-^¹ (T3), respectively.

The KMO test (≥0.6) and Bartlett’s sphericity test (*P* < 0.05) confirmed significant variable correlations, satisfying principal component analysis prerequisites (**Supplementary Table S4**). Principal component extraction criteria yielded two components with initial eigenvalues ≥1.69 and cumulative contribution rate ≥88.5% across years. Significant differences (*P* < 0.05) emerged in chemical component evaluations during curing stages between treatments over two years, with T3 demonstrating 140.97% increase and T1 of 179.24% decrease in composite scores relative to T2 treatment.

### 3.5 Effects of nitrogen regimes on chilling-induced polyphenol content

Nitrogen fertilization rates differentially modulated tobacco leaf polyphenol contents with significant changes (*P* < 0.05) observed at maturation stages of both years. Phenolic metabolite contents followed T1 > T2 > T3 for chlorogenic acid and rutin, while other biochemical indices remained statistically invariant across treatments. Over two consecutive years, throughout the curing stages, T1 exhibited elevated chlorogenic acid (+55.82%) and rutin (+38.65%) contents relative to T2, while T3 demonstrated reduced concentrations of chlorogenic acid (-10.82%) and rutin (-6.96%) compared to T2 treatment. At temperatures ranging from 25 to 48°C, the contents of chlorogenic acid and rutin upregulated. Chlorogenic acid contents ranged from 20.09 to 22.29 mg·g^-^¹ (T1), 9.75 to 16.46 mg·g^-^¹ (T2), and 8.33 to 12.94 mg·g^-^¹ (T3), with average synthesis rates of 0.02 mg·g^-^¹·h^-^¹ (T1), 0.06 mg·g^-^¹·h^-^¹ (T2), and 0.04 mg·g^-^¹·h^-^¹ (T3). Concurrently, rutin varied from 11.06 to 16.03 mg·g^-^¹ (T1), 6.60 to 12.51 mg·g^-^¹ (T2), and 5.07 to 11.15 mg·g^-^¹ (T3), exhibiting synthesis rates of 0.04 mg·g^-^¹·h^-^¹ (T1), 0.05 mg·g^-^¹·h^-^¹ (T2), and 0.05 mg·g^-^¹·h^-^¹ (T3), respectively.

The KMO test (≥0.54) and Bartlett’s sphericity test (*P* < 0.05) confirmed significant variable correlations, satisfying principal component analysis (PCA) prerequisites (**Supplementary Table S5**). Rutin was excluded from the 2020 dataset (KMO = 0.35) to eliminate multicollinearity. Principal component extraction yielded two components in 2019 (initial eigenvalues ≥1.5, cumulative contribution rate 73.0%) and three components in 2020 (initial eigenvalues ≥1.0, cumulative contribution rate 76.49%). Significant differences (*P* < 0.05) emerged in polyphenol contents across curing stages between treatments over two years, with T1 demonstrating increase (424.32%) and T3 decrease (250.74%) in composite scores relative to T2.

### 3.6 Effects of nitrogen regimes on chilling-induced antioxidant metabolism dynamics

**Figure 6:**
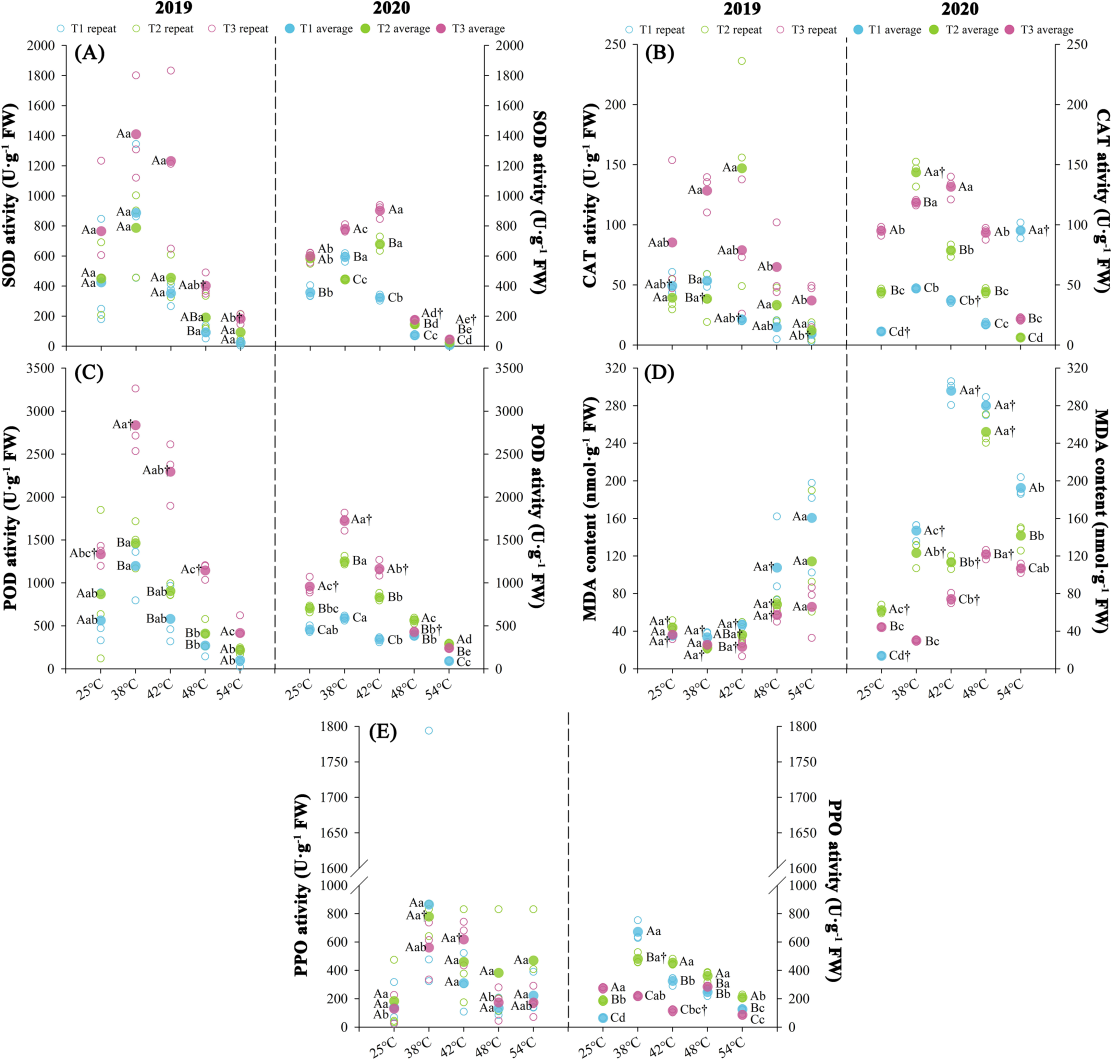
Effect of different N fertilization rates on antioxidant enzyme activities, i.e., SOD **(A)**, POD **(B)** CAT **(C)**, MDA **(D)**, and PPO activity **(E)** at different ambient air temperature during tobacco curing stage. Different capital letters indicated that there were significant differences between the same year, the same curing stage and different treatments (P < 0.05). Different lowercase letters indicated significant difference between the same year, different curing stages and same treatment (P < 0.05). “†” indicates significant differences between different years, curing stage, and treatments (P < 0.05).

Nitrogen fertilization rates differentially modulated antioxidant enzyme activities in tobacco leaves with significant variables (*P* < 0.05) observed at both experimental years with maturation stages (**Figure 6**). Antioxidant enzyme activities exhibited treatment-dependent gradients, such as SOD and POD followed the order T3 > T2 > T1 (*P* < 0.05), while CAT activity optimum in T3, with no significant difference between T2 and T1 (P > 0.1). Oxidative stress markers exhibited inverse patterns, with MDA accumulation ranked T1 > T2 > T3, and PPO activity lowest in T3. Over two consecutive years, throughout the curing stages, T3 exhibited enhanced SOD (+73.43%), POD (+64.54%), and CAT (+112.38%) activities relative to T2, while T1 demonstrated reduced SOD (-32.3%) and POD (-42.37%) activities. Concurrently, T3 showed decreased MDA (-31.22%) and PPO (-30.17%) levels compared to T2, with T1 displaying elevated MDA (+30.80%) content. At temperatures ranging from 25 to 48°C, SOD and POD activities exhibited initial increases followed by declines, with average change rates of 2.55 U·g^-^¹·h^-^¹ (T1), 2.88 U·g^-^¹·h^-^¹ (T2), and 3.25 U·g^-^¹·h^-^¹ (T3) for SOD, and 1.50 U·g^-^¹·h^-^¹ (T1), 2.47 U·g^-^¹·h^-^¹ (T2), and 2.96 U·g^-^¹·h^-^¹ (T3) for POD. Concurrently during the 25–54°C stage, MDA content displayed a continuous upward trend across treatments, with average change rates of 0.90 nmol·g^-^¹·h^-^¹ (T1), 0.44 nmol·g^-^¹·h^-^¹ (T2), and 0.27 nmol·g^-^¹·h^-^¹ (T3).

KMO test (≥ 0.67) and Bartlett’s sphericity test (*P* < 0.05) confirmed significant correlations among variables, satisfying principal component analysis (**Supplementary Table S6**). Principal component extraction identified two components across both years (initial eigenvalues ≥ 1.0, cumulative contribution rate ≥ 77.19%). Significant differences (*P* < 0.05) emerged in antioxidant enzyme assessment between treatments over the two years, with T3 demonstrating of 317.22% increase and T1 decrease (339.62%) in composite scores relative to T2 treatment.

### 3.7 Effects of nitrogen regimes on chilling-induced economic traits and sensory quality of cured tobacco

Nitrogen fertilization rates regimes influenced the visual quality of cured leaves (**Supplementary Figure S1**). Leaves treated with T3 exhibited better curing characteristics, including bright coloration, optimal lamina expansion, and minimal variegation or browning. In contrast, T2 and T1 treatments resulted in darker pigmentation, restricted leaf opening, conspicuous surface variegation, and dust accumulation effects that were most pronounced in T1 treatment.

Nitrogen fertilization rates regimes over two consecutive years significantly influenced the economic traits and sensory quality of cured leaves (*P* < 0.05) (**Table 1**). Across all economic traits and sensory quality indices, N regimes consistently ranked T3 > T2 > T1. Over two consecutive years, throughout the curing stages, T3 exhibited average gains compared to T2 of 7.35% (yield), 43.97% (economic value), 29.96% (proportion of middle and top-grade tobacco), 30.03% (average price), and 3.69% (sensory quality). Conversely, T1 exhibited marked reductions of 26.85, 52.41, 30.31, 34.91, and 12.56% in corresponding metrics relative to T2 treatment.

**Table 1:** Comprehensive evaluation of economic traits and sensory quality of cured tobacco leaves under different N fertilization rates.

Year	Treatment	Yield (kg·ha^-^¹)	Economic value (dollar·ha^-^¹)	Proportion of top and top-grade tobacco (%)	Average price (dollar·kg^-1^)	Sensory quality
2019	T1	1698.9 ± 39.36^B^	3483.26 ± 175.17^C,†^	44.99 ± 2.73^C^	2.05 ± 0.08^C^	70.5 ± 2.08^B^
	T2	2194.65 ± 106.3^A^	6841.82 ± 74.75^B^	59.21 ± 1.2^B^	3.12 ± 0.03^B^	83.5 ± 3.06^A^
	T3	2468.7 ± 113.63^A^	10423.84 ± 402.46^A^	81.56 ± 3.99^A,†^	4.22 ± 0.13^A^	87 ± 1.26^A^
2020	T1	1562.6 ± 25.23^B^	3152.35 ± 152.2^C,†^	40.2 ± 2.42^B^	2.16 ± 0.05^C^	71 ± 2.52^A^
	T2	2261.53 ± 62.02^A^	7120.58 ± 171.2^B^	63.42 ± 4^A^	3.35 ± 0.09^B^	78.5 ± 4.58^A^
	T3	2311.4 ± 99.51^A^	9654.37 ± 252.35^A^	77.48 ± 3.63^A,†^	4.18 ± 0.09^A^	81 ± 1.04^A^
Principal component analysis						
Indicator		F1 (Loading matrix)		Treatment	F _Comprehensive_	
		2019	2020		2019	2020
Yield (kg·ha^-^¹)		0.95	0.93	T1	-2.3C	-2.21^C^
Economic value (dollar·ha^-^¹)		1	0.99			
Proportion of top and top-grade tobacco (%)		0.97	0.94			
Average price (dollar·kg^-1^)		0.97	0.98	T2	0.14B	0.49^B^
Sensory quality		0.89	0.75			
Kaiser-Meyer-Olkin Measure of Sampling Adequacy. (Bartlett’s Test of Sphericity)		0.61 (*P* < 0.05)	0.69 (*P* < 0.05)			
Initial eigenvalues		4.56	4.25	T3	2.15A	1.72^A^
Contribution rate (%)		91.28	84.93			
Cumulative contribution rate (%)		91.28	84.93			

Data indicate mean average ± SE. Different letters indicate significant differences between different treatments in the same year (*P* < 0.05). “†” indicates that there are significant differences between the same treatments in different years (*P* < 0.05). T1, N 18.9 kg·ha^-1^ + Rot farm manure 15000 kg·ha^-1^; T2, N 27 kg·ha^-1^ + Rot farm manure 15000 kg·ha^-1^, which is the normal local N fertilizer rate; T3, N 35.1 kg·ha^-1^ + Rot farm manure 15000 kg·ha^-1^.

KMO test (≥ 0.61) and Bartlett’s sphericity test (*P* < 0.05) confirmed significant correlations among variables, satisfying principal component analysis (**Table 1**). Principal component extraction identified the first principal component across both years (initial eigenvalues ≥ 4.25, cumulative contribution rate ≥ 84.93%). Significant differences (*P* < 0.05) emerged in the composite scores of economic traits and sensory quality between treatments over two years with T3 demonstrating of 843.37% increase and T1 of 1146.94% decrease in composite scores relative to T2.

### 3.8 Effects of nitrogen regimes on tobacco quality and drying under chilling stress: PCA and stepwise regression analysis

Kaempferol-3-O-rutinoside exhibited negligible phytochemical-metabolic associations, compromising its utility in principal component extraction and stepwise regression modeling of cured tobacco quality traits (**Supplementary Figure S2**). Consequently, this variable was excluded from subsequent multivariate analyses. Multivariate analysis revealed two principal components explaining 84.29% cumulative variance in cured tobacco quality determinants (**Supplementary Table S7**). The rotated loading matrix (threshold >0.65) identified *F1* (60.5% variance) with N regime and photosynthetic pigments as principal determinants, opposed by reducing sugars, starch, and non-neochlorogenic polyphenols as secondary contributors. *F2* (23.8% variance) featured nicotine, total N, and neochlorogenic acid as primary drivers, contrasted by total sugars as secondary inhibitors.

Principal component factor scores (*F1*, *F2*) were derived from standardized indicator matrix multiplication (**Supplementary Table S8**). Subsequent factor analysis of economic traits and sensory quality attributes yielded a comprehensive evaluation factor (Y) for cured tobacco with KMO test measure of sampling adequacy at 0.73 (*P* < 0.05) and cumulative variance explanation rate of 87.53%. Stepwise regression modeling with *F1*/*F2* as predictors and Y as response variable generated the final Equation 4. Validation metrics confirmed statistical significance (adjusted *R*²=0.87, *P* < 0.01), non-collinearity (VIF<2.0), and residual independence/normality per Durbin-Watson/studentized residual diagnostics.


(4) Y=0.841F1+0.422F2

## 4 Discussion

### 4.1 Nitrogen fertilization modulates tissue structural integrity and biochemical homeostasis of fresh tobacco leaves under chilling stress

Leaf tissue architecture governs photosynthetic and transpiration efficiency, as well as stress acclimation, by structurally mediating physiological processes (Sun et al., 2024). Our field data demonstrate that N fertilization rates modulate chilling stress resilience in tobacco leaves by enhancing mesophyll development, particularly the formation of palisade tissue (**Figure 2**). This finding aligns with chilling stress-induced structural aberrations, including epidermal narrowing, palisade cell disorganization, increased intercellular porosity, and spongy tissue degradation (Bi et al., 2019). Nitrogen, as a key component of proteins, nucleic acids, and phospholipids, not only promotes the elongation and division of palisade tissue but also supports the expansion of spongy tissue (Yu et al., 2022). Higher N fertilization can sustain the long-term development of leaf tissue structure cells (Jiao et al., 2022). In this study, the ratio of palisade tissue to spongy tissue showed a negative correlation with the N fertilization rates (**Figure 2**). This may the stimulatory effect of increasing N fertilizer rates on spongy tissue was greater than palisade tissue. Alternatively, it may indicate that spongy tissue suffers less damage from chilling stress than palisade tissue (Jiang et al., 2025). Similarly, spongy tissue may experience less damage from chilling stress than palisade tissue (Dong et al., 2024).

Photosynthetic pigments serve as biomarkers for leaf maturation, photosynthetic performance, and organoleptic quality in tobacco leaves, with their catabolic derivatives constituting critical aroma precursors (Ren et al., 2023a). Our findings demonstrate that the N fertilization rates modulate chilling stress-induced pigment dynamics in field-grown tobacco, potentially by stabilizing thylakoid ultrastructure (**Figure 4**). This structural perturbation aligns with grana disassembly-induced photosystem II (PSII) dysfunction, wherein energy diversion mechanisms prioritize non-photochemical quenching over photochemical electron transport under suboptimal thermal regimes (Dhokne et al., 2022). Nitrogen modulates plastid pigment biosynthetic flux through enzymatic regulation of magnesium chelatase and phytoene synthase activities across fertilization gradients (Simkin et al., 2022; Hou et al., 2024). This metabolic regulation correlates with ultrastructural augmentation in tobacco leaf mesophyll cells, manifested through osmiophilic granule proliferation, chloroplast ontogeny, and thylakoid membrane biogenesis (Su et al., 2013). The chloroplast-localized deposition of pigment metabolites (Meng et al., 2024), the chlorophyll-enriched palisade tissue architecture under elevated N regimes provides a mechanistic basis for enhanced structural development and plastid pigment accrual observed in this study (**Figures 2**, **4**).

**Figure 7:**
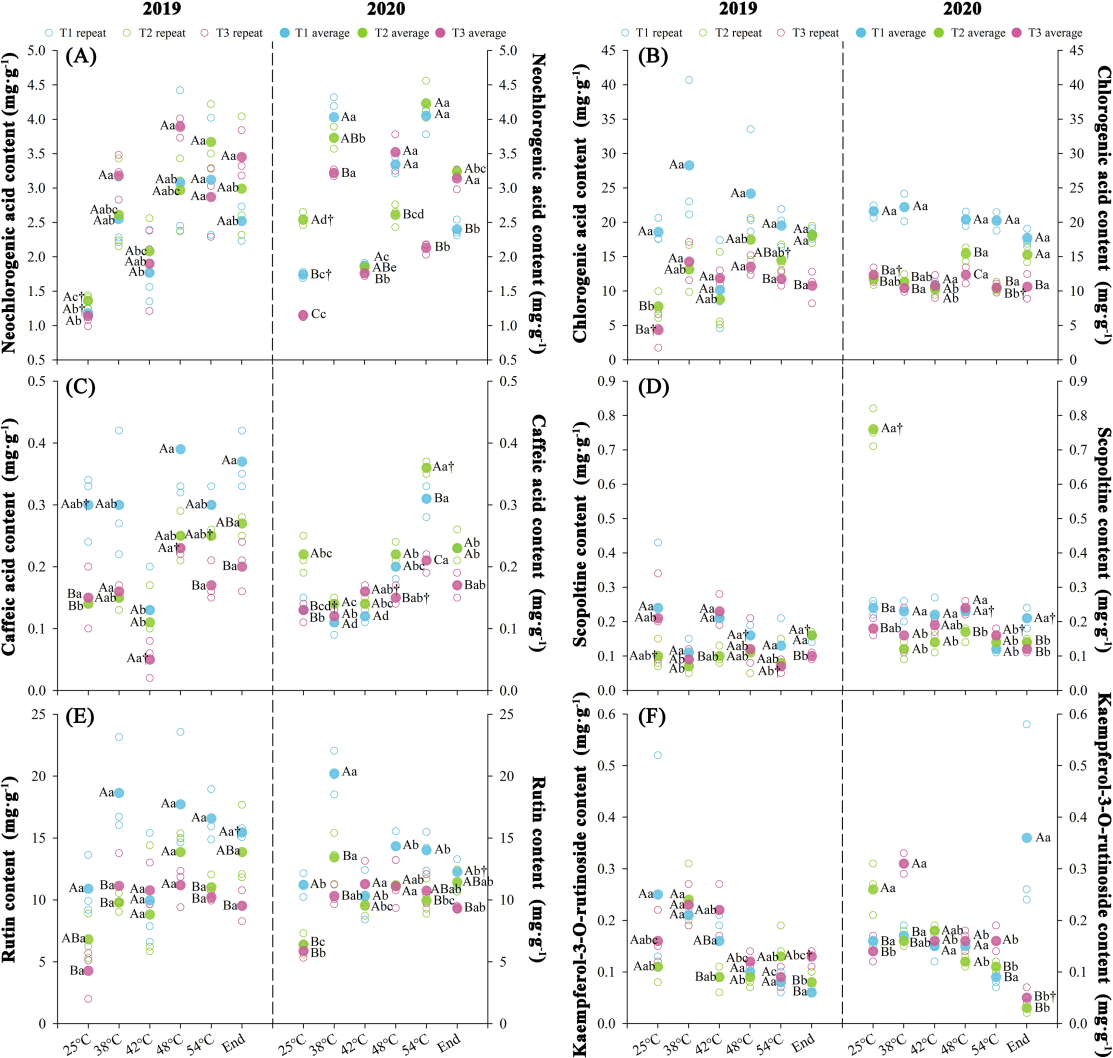
Effect of different N fertilization rates on neochlorogenic acid **(A)**, chlorogenic acid **(B)**, caffeic acid **(C)** scopoltine content **(D)** rutin content **(E)**, and kaempferol-3-O-rutinoside content **(F)** of tobacco during curing stages at different ambient air temperature. Different capital letters indicated that there were significant differences between the same year, the same curing stage and different treatments (P < 0.05). Different lowercase letters indicated significant difference between the same year, different curing stages and same treatment (P < 0.05). “†” indicates significant differences between different years, curing stage, and treatments (P < 0.05).

Chemical constituents serve as critical biometrics for evaluating organoleptic quality and phenotypic attributes in mature tobacco leaves (Li et al., 2024). Elevated N input in this study shifted C-N partitioning, reducing saccharide and starch accrual while enhancing alkaloid and total N concentrations (**Figure 5**). Chilling stress disrupts N metabolism through photoinhibitory PSII damage and differential modulation of enzymatic activities, promoting carbohydrate accumulation while impairing N assimilation efficiency via nitrate reductase and glutamine synthetase suppression (Kharbech et al., 2024; Feng et al., 2024a). This metabolic disequilibrium reflects the interdependent substrate provisioning between N-mediated protein synthesis and carbon-derived energy transduction, wherein reciprocal resource allocation mitigates chilling stress impacts (Huang et al., 2024; Shao et al., 2024). Nitrogen serves as the primary regulatory nexus in crop ontogeny, while chilling injury represents an exogenous stress during developmental phases (Liu et al., 2023). Our integrative assessment reveals that the elevated N fertilization rates enhances tissue ultrastructure, biochemical profiles, and antioxidant capacity in tobacco leaves under chilling stress (**Figures 2**, **4**, **5**), accompanied by reduced polyphenolic accumulation (**Figure 7**). Optimal N fertilization promotes mesophyll morphogenesis, photosynthetic quantum efficiency, and C-N metabolic flux equilibrium through optimization of antioxidant systems (Liu et al., 2025). In contrast, N deficiency exacerbates the effects of cold stress by causing thermodynamic disequilibrium in metabolic pathways (Farooq et al., 2017).

### 4.2 Nitrogen fertilization modulates water loss efficiency and biochemical homeostasis

Curing characteristics in tobacco leaves encompass curing resilience (leaf susceptibility to dehydration and chromogenesis) and curing amenability (stress tolerance during color fixation) (Chen et al., 2019a; Wu et al., 2020b). Our data demonstrate that chilling stress-induced water loss kinetics exhibit negative correlation with N input (**Figure 3**), while elevated N enhances chloroplast chlorophyll conversion capacity, antioxidant system robustness, and pigment metabolic flux (**Figures 4**, **6**). Successful curing necessitates kinetic coordination between dehydration and degreening rates to maintain hydrolytic homeostasis where optimal hydration preserves enzymatic activity for aroma biogenesis, whereas accelerated desiccation disrupts pigment metabolism and phytochemical transformation (Chen et al., 2020; Wu et al., 2020a).

Suboptimal dehydration kinetics during color fixation trigger widespread enzymatic browning in tobacco leaves (Chen et al., 2019b). This study establishes that elevated N fertilization rates optimizes water loss regulation (**Figure 3**), aligning with ideal curing-phase hydric dynamics to facilitate photosynthetic pigment catabolism and phytochemical transformation (Meng et al., 2024). Enhanced N regimes promote mesophyll histogenesis that prolongs dehydration kinetics while reinforcing antioxidant-mediated membrane stabilization under chilling stress. Consequently, accelerated plastid pigment turnover coordinates efficient chromogenesis and aromatic compound biosynthesis through synchronized degreening-dehydration coupling (**Figure 4**), concurrently preserving photosynthetic apparatus integrity and physiological homeostasis during chilling exposure.

The antioxidant system constitutes a critical redox homeostasis indicator for plant stress acclimation (Zuo et al., 2025), where superoxide dismutase, peroxidase, and catalase collectively mitigate oxidative damage. Elevated N regimes in this study maximized antioxidant enzymatic kinetics while minimizing malondialdehyde accrual (**Figure 6**), demonstrating superior reactive oxygen species (ROS) scavenging capacity and plasma membrane integrity preservation under chilling stress. Concurrently, attenuated polyphenol oxidation rates during curing phases indicate N-mediated suppression of enzymatic browning, as cryoinhibition of polyphenol oxidase (PPO) activity necessitates precise curing environment modulation for controlled phenolic metabolism (Zhang et al., 2023b). This integrated antioxidant response counters ROS-induced membrane peroxidation cascades that disrupt photosynthetic electron transport, respiratory energetics, and hydric equilibrium ultimately preventing chlorophyll degradation, tissue necrosis, and metabolic collapse (Gholizadeh et al., 2024). Through optimized N allocation, enhanced enzymatic robustness maintains thermodynamic equilibrium in plant defense systems under abiotic perturbation.

### 4.3 Nitrogen fertilization modulates economic traits and sensory quality of cured tobacco leaves

Nitrogen fertilization modulates tobacco agroeconomic optimization through regulated resource allocation that enhances yield-caliber equilibrium (Wang et al., 2024). Our findings demonstrate superior economic trait integration and organoleptic quality in high-N regimes (**Table 1**), corroborating established N-driven yield escalation mechanisms where leaf biomass, market value, and premium-grade proportions exhibit positive dose-response relationships (Zhu et al., 2024). This metabolic steering elevates metabolites (total N/nicotine) while suppressing carbohydrate accrual (total/reducing sugars) through carbon-N metabolic antagonism, a phenomenon mechanistically rooted in reductant competition during ammonia assimilation (Santiago and Tegeder, 2017; Zhao et al., 2023). Notably, such N-mediated chilling stress resilience aligns with photosynthetic apparatus fortification observed in Poaceae species under chilling stress (Soualiou et al., 2023). In contrast, N-deficit conditions amplify carbon flux via enhanced amylolytic activity and starch deposition (Li et al., 2018), underscoring the redox-homeostasis-dependent trade-off between N assimilation efficiency and carbon metabolic vigor that governs phytochemical profiles and aroma biogenesis (Zhan et al., 2021; Hao et al., 2022; Ma et al., 2024).

Polyphenols function as phenylpropanoid metabolites and aroma precursors, critically defining tobacco quality through biochemical interactions of their dominant constituents (Liu et al., 2022a). Elevated N regimes reduced chlorogenic acid and rutin accumulation in tobacco leaves, whereas chilling stress activates phenylpropanoid pathways via phenylalanine ammonia-lyase (PAL) and polyphenol oxidase (PPO) induction through enzymatic potentiation and transcriptional upregulation (Khaliq et al., 2023). N-mediated suppression of polyphenol biosynthesis through phenylpropanoid pathway regulation concurrently enhances reactive oxygen species scavenging capacity (Rao and Zheng, 2025), aligning with documented negative correlations between N input and chlorogenic acid/rutin/scopolamine concentrations attributable to transcriptional downregulation of core phenylpropanoid genes, such as *PAL, 4CL, CHS, CHI* (Niu et al., 2017; Zhang et al., 2017; He et al., 2019). This trade-off facilitates antioxidant system fortification and membrane integrity preservation under chilling stress while mitigating enzymatic browning risks, ultimately optimizing cured leaf quality.

Integrated correlation analysis, principal component analysis, and multiple linear stepwise regression identified N fertilization rates and photosynthetic pigments content as primary positive determinants of economic traits and sensory quality in chilling stressed tobacco leaves, with N metabolites as secondary contributors, while sugar derivatives and polyphenols constituted key negative modulators (**Supplementary Table S8**). Elevated N regimes directly enhance mesophyll histogenesis, photosynthetic quantum yield, and C-N metabolic flux equilibrium while mitigating cryoinhibition of antioxidant enzymatic kinetics and malondialdehyde accrual. This metabolic steering concurrently alleviates membrane thermo-hydric disintegration during flue-curing through controlled dehydration-chromogenesis coordination. Although saccharide and phenolic accrual confer marginal quality enhancement, their efficacy remains substantially inferior to N-mediated systemic optimization. Consequently, altitudinal agronomic N augmentation in Yunnan tobacco systems ensures yield stability and quality preservation under field chilling stress through integrated photo-biochemical pathway modulation.

## 5 Conclusions

Field chilling stress in Yunnan, China high-altitude tobacco systems compromises leaf quality parameters during pre- and post-harvest phases. However, 30% enhancement in N fertilization rates effectively mitigates these effects by preserving histological integrity and maintaining the balance of moisture and physiobiochemical processes throughout curing. Elevated N regimes under chilling stress maintain histological structure, reduce water loss during curing, enhance plastid pigment turnover, and increase the accumulation of nitrogenous compounds (total nitrogen and nicotine), while also strengthening antioxidant capacity. These combined effects optimize the organoleptic quality of cured leaves despite concurrent reductions in carbon metabolites and polyphenolic content. Multivariate analyses identified N dosage and chloroplast pigment dynamics as primary determinants of quality with nicotine and neochlorogenic acid serving as secondary modulators. Strategic 30% N supplementation in high-altitude tobacco cultivation emerges as an effective adaptation strategy to chilling stress, preserving yield quality and biochemical integrity during critical phenophases in August. Future research should prioritize multi-omics analyses of N-mediated chloroplast biogenesis mechanisms, combined with precision fertilization modeling and long-term agroecological assessments, to enhance sustainable tobacco production frameworks amid climate variability.

## Data Availability

The original contributions presented in the study are included in the article/**Supplementary Material**. Further inquiries can be directed to the corresponding author/s.
