# Supplementary material for: Nitrogen fertilization rates affect quality and curing characteristics of tobacco during the harvesting period under field chilling stress
Source: Front Plant Sci. 2025 Oct 29;16:1681963. doi: 10.3389/fpls.2025.1681963 (PMC12605925; doi:10.3389/fpls.2025.1681963)
Supplement: Supplementary file 1 [file DataSheet1.docx]

**Title page**

**Types of paper:** Regular papers

**Title:** Nitrogen fertilization rates affect quality and curing characteristics of tobacco during the harvesting period under field chilling stress

**Author names and affiliations:** Ke Ren ^1,^ †, Zehui Wei ^2,^ †, Kaiyuan Gu ^3^, Guorun Fu ^4^, Long Zhang ^4^, Hong Zhang ^4^, Bin Zhou ^4^, Feng Chen ^4^, Yi Chen ^1^, Khanom Simarani ^2,^ *, Binbin Hu ^1,^ **

^1^ Yunnan Academy of Tobacco Agricultural Sciences, Kunming, Yunnan 650021, China

^2^ Division of Microbiology, Institute of Biological Sciences, Faculty of Science, Universiti Malaya, 56100 Kuala Lumpur, Malaysia.

^3^ College of Agronomy and Biotechnology, Engineering Research Center of South Upland Agriculture, Southwest University, Ministry of Education, Chongqing 400715, China

^4^ Chuxiong Tobacco Bureau of Yunnan Province, Chuxiong 675000, China

*** Corresponding author**. Division of Microbiology, Institute of Biological Sciences, Faculty of Science, Universiti Malaya, 56100 Kuala Lumpur, Malaysia.

**** Corresponding author**. Yunnan Academy of Tobacco Agricultural Sciences, Kunming 650021, China

**E–mail addresses:** hanom_ss@um.edu.my (K. Simarani), hubinbin20072008@163.com (B. Hu)

† Ke Ren and Zehui Wei contributed equally to this work.

**Number of words:** 8014 words in the main text

**Number of figures and tables:** 1 table and 7 figures

**Supporting information:** 8 tables and 2 figures

**2.6 Data Analysis**

The specific data processing steps are divided into three parts (Pearson correlation analysis, principal component analysis and multiple linear stepwise regression) as follows:

**Part one: Pearson correlation analysis**

(1) Normality test: the data obey normal distribution; and passes Levene’s test of homogeneity of variance and Shapiro-Wilk test of normal distribution of residences. If the preconditions of ANOVA such as normal distribution and homogeneity of variance are not satisfied, the skewness was judged by its skewness value and then logarithmic conversion was carried out.

(2) Solve the linear correlation coefficient. Through Pearson product moment correlation formula calculation (puth et al.,2014), it was confirmed that there was significant correlation among most indicators (*P* < 0.05) The linear regression and correlation formula (1-1) was shown below:

$r_{xy}=\frac{\sum_{i=1}^{n} (x_{i}-\bar{x})(y_{i}-\bar{y})}{\sqrt{\sum_{i=1}^{n} {(x_{i}-\bar{x})}^{2}\sum_{i=1}^{n} {(y_{i}-\bar{y})}^{2}}}$ (1-1)

Where $\bar{x}$、$\bar{y}$ are the averages of the variables x and y, respectively. $i$ is the observed value.

**Part two: principal component analysis**

(1) After the normality test of the data for the same category of indicators was carried out above, Z-score was used to eliminate the dimension to explain the covariance structure of the original variable. The formula (1-2) was shown below:

$Z_{i}=\frac{x_{i}-\bar{x}}{s}$ (1-2)

Where $x_{i}$is the first observed value of the variable, $\bar{x}$ is the mean of the all-measured values for variable $x$, and$s$ is the standard deviation of the variable $x$.

(2) Furthermore, factor analysis was used to determine the Kaiser-Meyer-Olkin (KMO) Measure of Sampling > 0.5, and Bartlett's Test of Sphericity concomitant probability *P* < 0.05, indicating that all data in this study can be used for principal component analysis after testing. However, for the KMO test < 0.5, abnormal variables need to be eliminated or other variables associated with abnormal variables need to be introduced. In this study, variables with high weight among the principal components whose eigenvalue was close to 0 are eliminated, and then the remaining variables are used for principal component analysis again, until the KMO value meets the general conditions. Initial Characteristic values >1; Cumulative >80%. The principle was applied for screening the number of principal components in each analysis category.

(3) Solve the root of the characteristic equation. The contribution rate of the variance of the $i$th principal component is redistributed in the total variance. The formula (1-3) was shown below:

$\lambda_{i}=\frac{\sum_{i=1}^{m} {(p_{i}-\bar{p}_{i})}^{2}}{n-1}$ (1-3)

In the formula,$\lambda_{i}$is the$i$th characteristic value; $m$is the variable; $p_{i}$ is the difference of n observed objects studied by the original variable, which is a linear function used to represent the original variable.

(4) Solve the cumulative contribution rate of the first $k$principal components. First, by solving the percentage of the information contained in each component in the total information, that is, the percentage of the variance provided by each component in the total variance, and then summing up, the formula (1-4) was shown below:

$\sum_{i=1}^{k} \frac{\lambda_{i}}{\sum_{i=1}^{m} \lambda_{i}}=\sum_{i=1}^{k} \frac{\lambda_{i}}{m}$ (1-4)

(5) Solve the weight coefficient of the principal component corresponding to each variable.

The formula (1-5) was shown below:

$u_{m}=\frac{a_{ij}}{\sqrt{\lambda_{i}}}$,$i,j=1,\cdot\cdot\cdot,m$ (1-5)

In the formula, the load $a_{ij}$ is the correlation coefficient of the $i$th component and the $j$ variable.

(6) Solve the principal component scores. The formula (1-6) was shown below:

$F_{m}=\sum_{i}^{k} Z_{i}\times u_{m}$ (1-6)

(7) Solve the comprehensive principal component fraction. The formula (1-7) was shown below:

$F_{Comprehensive}=\frac{\lambda_{i}}{\sum_{i=1}^{m} \lambda_{i}}\times F_{m}$ (1-7)

**Part three: Multiple linear stepwise regression**

(1) A principal component stepwise regression model was established based on the indicators of initially flue-cured tobacco leaves nitrogen fertilization rates, plastid pigment, conventional chemical substances and polyphenols were selected as independent variables. Economic traits and sensory quality were comprehensive dependent variables. Principal component analysis was used to analyze variance and extract common factors. Quartimax method was used to orthogonally rotate the factor loading matrix to explain the factor components. Finally, according to the principal component score coefficient matrix, the stepwise regression model of the economic traits of initially flue-cured tobacco leaves and the factors of initially flue-cured tobacco leaves directly cured by tobacco growers was established. The model equation was shown in (1-8) (Zhan et al.,2013).

$Y=\beta_{0}+\beta_{1}x_{1}+\beta_{2}x_{2}+\cdot\cdot\cdot+\beta_{p}x_{p}+\varepsilon$,$E(\varepsilon)=0,D(\varepsilon)=\sigma^{2}$ (1-8)

Where, ε is a random error, $\beta_{0}$ is a regression constant, $\beta_{1}{、\beta}_{2}\cdot\cdot\cdot,\beta_{p}$is a regression coefficient, and $x_{1}{、x}_{2}\cdot\cdot\cdot,x_{p}$ is a variable.

Where, $E\left( \varepsilon\right)$is its mathematical expectation and $D(\varepsilon)$ is its variance.

(2) The F significance level was used to control the stepping. The standard of entering the regression model was < 0.05, and the standard of excluding the regression model was > 0.1.

(3) $\mathrm{Adjusted}R^{2}$ is the coefficient of determination. The coefficient is to eliminate the illusion that the number of variables is too high due to the blind increase of the number of variables. And it is an important index of multivariate linear goodness of fit. The equation was shown in (1-9).

$\mathrm{Adjusted}R^{2}=1-\frac{\sum\left( y-\hat{y} \right)^{2}/(n-k-1)}{\sum\left( y-\bar{y} \right)^{2}/(n-1)}$ (1-9)

In the formula, $k$ is the number of independent variables, $n$is the number of observations, $y$ is the measured value, $\hat{y}$ is the predicted value, and $\bar{y}$ is the average of the measured value.

(4) Durbin-Watson and residual normal distribution test. Durbin-Watson test was used to diagnose the independence of residuals, its value range is 0 < D < 4, when the value is approximately = 2, the residuals are independent of independent variables. In addition, the Shapiro-Wilk normal distribution test is carried out on the unstandardized residuals and biochemical residuals to comprehensively determine the final fitting effect of the model.

**Supplementary Tables and Figures**

**Table captions:**

**Table S1** The main curing method for bulk-curing barns in Laojun Mountain Town

**Table S2.** Principal component analysis matrix and comprehensive evaluation of leaf tissue structure under different nitrogen fertilization rates.

**Table S3.** Principal component analysis matrix and comprehensive evaluation of tobacco plastid pigment under different nitrogen rates.

**Table S4.** Principal component analysis matrix and comprehensive evaluation of conventional chemical components of tobacco leaves under different nitrogen fertilization rates.

**Table S5.** Principal component analysis matrix and comprehensive evaluation of polyphenols in tobacco leaves under different nitrogen fertilization rates.

**Table S6.** Principal component analysis matrix and comprehensive evaluation of antioxidant enzyme activities in tobacco leaves under different nitrogen fertilization rates.

**Table S7.** Principal Component Analysis and Post Rotation Load Matrix of Economic Traits and Sensory Smoking Control Elements of Flue-cured Tobacco.

**Table S8.** Model summary, Analysis of variance and coefficient of initially flue-cured tobacco leaves.

**Figure Captions:**

**Figure S1.** Comparison of appearance of flue-cured tobacco after curing with different N fertilization rates.

**Figure S2.** Correlation analysis of factors which control economic traits of flue-cured tobacco leaves.

**TABLE S1** The main curing method for bulk-curing barns in Laojun Mountain Town.

| Index | Start | Yellowing stage | | | Color fixing stage | | | Stem drying stage |
| --- | --- | --- | --- | --- | --- | --- | --- | --- |
| Dry bulb temperature (℃) | 25 | 34 | 38 | 42 | 48 | 54 | 54 | 66 |
| Wet bulb temperature (℃) | 20 | 32 | 36 | 37 | 37 | 37 | 37.5 | 38.5 |
| Curing time (h) | 0 | 19 | 25 | 18 | 15 | 22 | 2 | 30 |

**TABLE S2** Principal component analysis matrix and comprehensive evaluation of leaf tissue structure under different nitrogen fertilization rates.

| Indicator | F1 (Loading matrix) | | Treatment | F _Comprehensive_ | |
| --- | --- | --- | --- | --- | --- |
|  | 2019 | 2020 |  | 2019 | 2020 |
| TUE | 0.91 | 0.89 | T1 | -2.42C | -2.05C |
| PTT | 0.86 | 0.9 |  |  |  |
| STT | 0.99 | 0.98 |  |  |  |
| TLE | 0.83 | 0.91 |  |  |  |
| BT | 0.99 | 0.97 | T2 | 0.44B | -0.03B |
| PTT/STT | -0.95 | -0.77 |  |  |  |
| Kaiser-Meyer-Olkin Measure of Sampling Adequacy. (Bartlett’s Test of Sphericity) | 0.6 (*P*<0.05) | 0.70 (*P*<0.05) |  |  |  |
| Initial Eigenvalues | 5.12 | 4.95 | T3 | 1.97A | 2.08A |
| Contribution rate (%) | 85.35 | 82.27 |  |  |  |
| Cumulative contribution rate (%) | 85.35 | 82.27 |  |  |  |

Note: TUE, Thickness of upper epidermis; PTT, Palisade tissue thickness; STT: Sponge tissue thickness; TLE: Thickness of lower epidermis; BT: Blade thickness; PTT/STT: Palisade tissue thickness/Sponge tissue thickness. T1, N 18.9 kg·ha^-1^ + Rot farm manure 15000 kg·ha^-1^; T2, N 27 kg·ha^-1^ + Rot farm manure 15000 kg·ha^-1^, which is the normal local nitrogen fertilizer rate; T3, N 35.1 kg·ha^-1^ + Rot farm manure 15000 kg·ha^-1^. The same as below.

**TABLE S3** Principal component analysis matrix and comprehensive evaluation of tobacco plastid pigment under different nitrogen rates.

| Indicator | Load matrix of the principal components | | | |
| --- | --- | --- | --- | --- |
|  | F1 | | F2 | |
|  | 2019 | 2020 | 2019 | 2020 |
| Chlorophyll a | 0.73 | 0.96 | 0.62 | -0.09 |
| Chlorophyll b | 0.74 | 0.96 | 0.61 | 0.03 |
| Lutein | 0.83 | 0.05 | -0.52 | 0.95 |
| β-carotene | 0.78 | 0.01 | -0.6 | 0.95 |
| Kaiser-Meyer-Olkin Measure of Sampling Adequacy. (Bartlett's Test of Sphericity) | —— | | 0.53 (*P*<0.05) | 0.50 (*P*<0.05) |
| Initial Eigenvalues | 2.38 | 1.84 | 1.39 | 1.82 |
| Contribution rate (%) | 59.5 | 45.93 | 34.67 | 45.44 |
| Cumulative contribution rate (%) | 59.5 | 45.93 | 94.17 | 91.36 |
| Treatment | Sampling temperature（°C） | | F _Comprehensive_ | |
|  |  |  | 2019 | 2020 |
| T1 | 25 | | -0.41Aa | -0.66Bb |
|  | 38 | | 0.05Aa | -0.13Ba |
|  | 42 | | -0.59Aa | -0.68Bb |
|  | 48 | | -0.7Ba† | -1.05Cc† |
|  | 54 | | -0.73Aa† | -1.1Cc† |
|  | End | | -0.65Ba | -0.92Cbc |
| T2 | 25 | | 2.4Aa | 1.73Aa |
|  | 38 | | 0.01Aa | -0.14Bb |
|  | 42 | | -0.33Aa | -0.25Abc |
|  | 48 | | -0.43Ba | -0.43Bc |
|  | 54 | | -0.15Aa | -0.03Bb |
|  | End | | -0.42ABa | -0.37Bc |
| T3 | 25 | | 1.91Aa | 1.6Aa |
|  | 38 | | 0.28Aa | 0.18Ac |
|  | 42 | | -0.52Aa | -0.54Bd |
|  | 48 | | 0.45Aa | 1.08Ab |
|  | 54 | | -0.35Aa | 0.39Ac |
|  | End | | 0.2Aa† | 1.31Aab† |

Note: Different capital letters indicated that there were significant differences between the same year, the same curing stage and different treatments (*P* < 0.05). Different lowercase letters indicated significant difference between the same year, different curing stages and the same treatment (*P* < 0.05). “†” indicates significant differences between different years, same curing stage, and same treatments (*P* < 0.05). The same as below.

**TABLE S4** Principal component analysis matrix and comprehensive evaluation of conventional chemical components of tobacco leaves under different nitrogen fertilization rates.

| Indicator | Load matrix of the principal components | | | |
| --- | --- | --- | --- | --- |
|  | F1 | | F2 | |
|  | 2019 | 2020 | 2019 | 2020 |
| Total sugar | 0.85 | 0.92 | -0.49 | -0.36 |
| Reducing sugar | 0.72 | 0.77 | -0.65 | -0.59 |
| Total nitrogen | 0.41 | 0.34 | 0.82 | 0.81 |
| Nicotine | 0.35 | 0.55 | 0.81 | 0.74 |
| Starch | -0.87 | -0.94 | -0.31 | -0.1 |
| Kaiser-Meyer-Olkin Measure of Sampling Adequacy. (Bartlett's Test of Sphericity) | —— | | 0.61 (*P*<0.05) | 0.60 (*P*<0.05) |
| Initial Eigenvalues | 2.3 | 2.73 | 2.1 | 1.69 |
| Contribution rate (%) | 45.94 | 54.61 | 41.92 | 33.89 |
| Cumulative contribution rate (%) | 45.94 | 54.61 | 87.86 | 88.5 |
| Treatment | Sampling temperature（°C） | | F _Comprehensive_ | |
|  |  |  | 2019 | 2020 |
| T1 | 25 | | -2.25Bb | -2.52Bc |
|  | 38 | | -0.21Ba | -0.34Cab |
|  | 42 | | -0.05Aa | -0.02Ba |
|  | 48 | | -0.2Ba | -0.12Bab |
|  | 54 | | -0.52Aab | -0.64Cb |
|  | End | | -0.43Ba | -0.54Bab |
| T2 | 25 | | -0.97Ab† | -1.68Ac† |
|  | 38 | | 0.4ABab | 0.63Ba |
|  | 42 | | 0.25Aab | 0.73Aa |
|  | 48 | | 0.14ABab | 0.4ABab |
|  | 54 | | 0.29Aab | 0.2Bb |
|  | End | | 0.62ABa | 0.55Aab |
| T3 | 25 | | -0.93Ab | -1.08Ad |
|  | 38 | | 1.15Aa | 1.58Aa |
|  | 42 | | 0.01Aab | 0.42Ac |
|  | 48 | | 1.25Aa | 0.72Abc |
|  | 54 | | 0.7Aab | 0.95Ab |
|  | End | | 0.75Aa | 0.78Abc |

**TABLE S5** Principal component analysis matrix and comprehensive evaluation of polyphenols in tobacco leaves under different nitrogen fertilization rates.

| Indicator | Load matrix of the principal components | | | | |
| --- | --- | --- | --- | --- | --- |
|  | F1 | | F2 | | F3 |
|  | 2019 | 2020 | 2019 | 2020 | 2020 |
| Neochlorogenic acid | 0.62 | 0.76 | -0.38 | 0.32 | -0.09 |
| Chlorogenic acid | 0.93 | 0.34 | 0.26 | 0.42 | 0.82 |
| Caffeic acid | 0.86 | 0.65 | 0.02 | 0.39 | -0.47 |
| Scopoltine | -0.1 | -0.46 | 0.85 | 0.69 | -0.17 |
| Rutin | 0.93 | —— | 0.18 | —— | —— |
| Kaempferol-3-O-rutinoside | -0.14 | -0.6 | 0.73 | 0.54 | -0.03 |
| Kaiser-Meyer-Olkin Measure of Sampling Adequacy. (Bartlett's Test of Sphericity) | —— | | 0.56 (*P*<0.05) | —— | 0.54 (*P*<0.05) |
| Initial Eigenvalues | 2.88 | 1.69 | 1.5 | 1.2 | 1.0 |
| Contribution rate (%) | 48.07 | 33.83 | 24.93 | 24.05 | 18.61 |
| Cumulative contribution rate (%) | 48.07 | 33.83 | 73 | 57.88 | 76.49 |
| Treatment | Sampling temperature（°C） | | | F _Comprehensive_ | |
|  |  |  |  | 2019 | 2020 |
| T1 | 25 | | | 0.53Aa | 0.16Ab |
|  | 38 | | | 1.36Aa | 0.78Aa |
|  | 42 | | | -0.48Aa | -0.67Ac |
|  | 48 | | | 1.32Aa | 0.67Aa |
|  | 54 | | | 0.72Aa | 1.14Aa |
|  | End | | | 0.73Aa | 0.19ABb |
| T2 | 25 | | | -1.09Ba† | -0.24Cc† |
|  | 38 | | | -0.36Aa | -0.08Bbc |
|  | 42 | | | -0.94Aa | -0.68Ad |
|  | 48 | | | 0.28Aa | 0.14Babc |
|  | 54 | | | 0.03Ba | 0.54Ba |
|  | End | | | 0.45Aa | 0.38Aab |
| T3 | 25 | | | -1.16Ba | -0.73Bd |
|  | 38 | | | -0.12Aa | -0.39Cbc |
|  | 42 | | | -0.4Aa | -0.61Acd |
|  | 48 | | | 0.04Aa | -0.03Ba |
|  | 54 | | | -0.52Ca | -0.42Cbc |
|  | End | | | -0.37Ba | -0.17Bab |

**TABLE S6** Principal component analysis matrix and comprehensive evaluation of antioxidant enzyme activities in tobacco leaves under different nitrogen fertilization rates.

| Indicator | Load matrix of the principal components | | | |
| --- | --- | --- | --- | --- |
|  | F1 | | F2 | |
|  | 2019 | 2020 | 2019 | 2020 |
| SOD | 0.890 | 0.900 | 0.130 | 0.060 |
| POD | 0.900 | 0.930 | -0.030 | 0.070 |
| CAT | 0.660 | 0.780 | -0.560 | 0.150 |
| MDA | -0.760 | -0.660 | 0.140 | 0.600 |
| PPO | 0.490 | 0.180 | 0.800 | 0.920 |
| Kaiser-Meyer-Olkin Measure of Sampling Adequacy. (Bartlett's Test of Sphericity) | —— | | 0.76 (*P*<0.05) | 0.67 (*P*<0.05) |
| Initial Eigenvalues | 2.86 | 2.76 | 1 | 1.23 |
| Contribution rate (%) | 57.21 | 55.19 | 19.98 | 24.58 |
| Cumulative contribution rate (%) | 57.21 | 55.19 | 77.19 | 79.77 |
| Treatment | Sampling temperature（°C） | | F _Comprehensive_ | |
|  |  |  | 2019 | 2020 |
| T1 | 25 | | -0.33Aab | -0.76Cbc |
|  | 38 | | 0.93Aa | 0.67Ba |
|  | 42 | | -0.34Babc | -0.58Cb |
|  | 48 | | -1.12Bbc | -1.05Cd |
|  | 54 | | -1.44Ac | -0.93Ac |
| T2 | 25 | | -0.22Aab | 0.03Bc |
|  | 38 | | 0.91Aa | 1.35Aa |
|  | 42 | | 0.34ABab | 0.84Bb |
|  | 48 | | -0.52ABab | -0.45Bd |
|  | 54 | | -0.86Ab | -1.17Be |
| T3 | 25 | | 0.26Abc | 0.73Ac |
|  | 38 | | 1.83Aa | 1.57Aa |
|  | 42 | | 1.46Aab | 1.17Ab |
|  | 48 | | -0.18Acd | -0.19Ad |
|  | 54 | | -0.71Ad† | -1.24Be† |

**TABLE S7** Principal component analysis and post rotation load matrix of economic traits and sensory quality control elements of flue-cured tobacco.

| Principal component analysis and eigenvalues | | Factor | |
| --- | --- | --- | --- |
|  |  | 1 | 2 |
| Initial Eigenvalues | Eigenvalues | 10.39 | 2.26 |
|  | Contribution rate (%) | 69.23 | 15.05 |
|  | Cumulative contribution rate (%) | 69.23 | 84.29 |
| Rotation sums of squared loadings | Eigenvalues | 9.08 | 3.56 |
|  | Contribution rate (%) | 60.53 | 23.75 |
|  | Cumulative contribution rate (%) | 60.53 | 84.29 |
| Kaiser-Meyer-Olkin Measure of Sampling Adequacy. (Bartlett's Test of Sphericity) | 0.70 (*P*＜0.05) | | |
| Rotated load matrix of the principal components | | Factor | |
|  |  | 1 | 2 |
| Pure nitrogen | | 0.90 | 0.29 |
| Chlorophyll a | | 0.95 | 0.10 |
| Chlorophyll b | | 0.92 | -0.19 |
| Lutein | | 0.92 | 0.09 |
| β-carotene | | 0.90 | 0.38 |
| Total sugar | | -0.52 | -0.69 |
| Reducing sugar | | -0.69 | -0.65 |
| Total nitrogen | | 0.53 | 0.76 |
| Nicotine | | 0.43 | 0.86 |
| Starch | | -0.78 | -0.52 |
| Neochlorogenic acid | | 0.50 | 0.79 |
| Chlorogenic acid | | -0.95 | 0.08 |
| Caffeic acid | | -0.80 | -0.07 |
| Scopoltine | | -0.74 | -0.32 |
| Rutin | | -0.83 | 0.26 |

**TABLE S8** Model summary, Analysis of variance and coefficient of initially flue-cured tobacco leaves.

| Model Summary and ANOVA | | | | | |
| --- | --- | --- | --- | --- | --- |
| Variable | | | | 1 | 2 |
| Adjusted *R*^2^ | | | | 0.69 | 0.87 |
| Durbin-Watson | | | | —— | 2.23 |
| Sum of Squares | | Regression | | 12.01 | 15.04 |
|  |  | Residual | | 4.96 | 1.93 |
| Mean Square | | Regression | | 12.01 | 7.52 |
|  |  | Residual | | 0.31 | 0.13 |
| F | | | | 38.75 | 58.31 |
| *P* | | | | ＜0.05 | |
| Coefficients | | | | | |
| Model | 1 | | 2 | | |
|  | (Constant) | *F1* | (Constant) | *F1* | *F2* |
| Unstandardized Coefficients | 0 | 0.84 | 0 | 0.84 | 0.42 |
| Standard Error | 0.13 | 0.14 | 0.09 | 0.09 | 0.09 |
| Standardized Coefficients | —— | 0.84 | —— | 0.84 | 0.42 |
| t | 0 | 6.23 | 0.01 | 9.65 | 4.84 |
| *P* | 1 | 0 | 1 | 0 | 0 |
| Tolerance | —— | 1 | —— | 1 | 1 |
| VIF | —— | 1 | —— | 1 | 1 |
| Unstandardized Residual | Shapiro-Wilk | 0.76 | | | |
| Studentized Residual |  | 0.75 | | | |


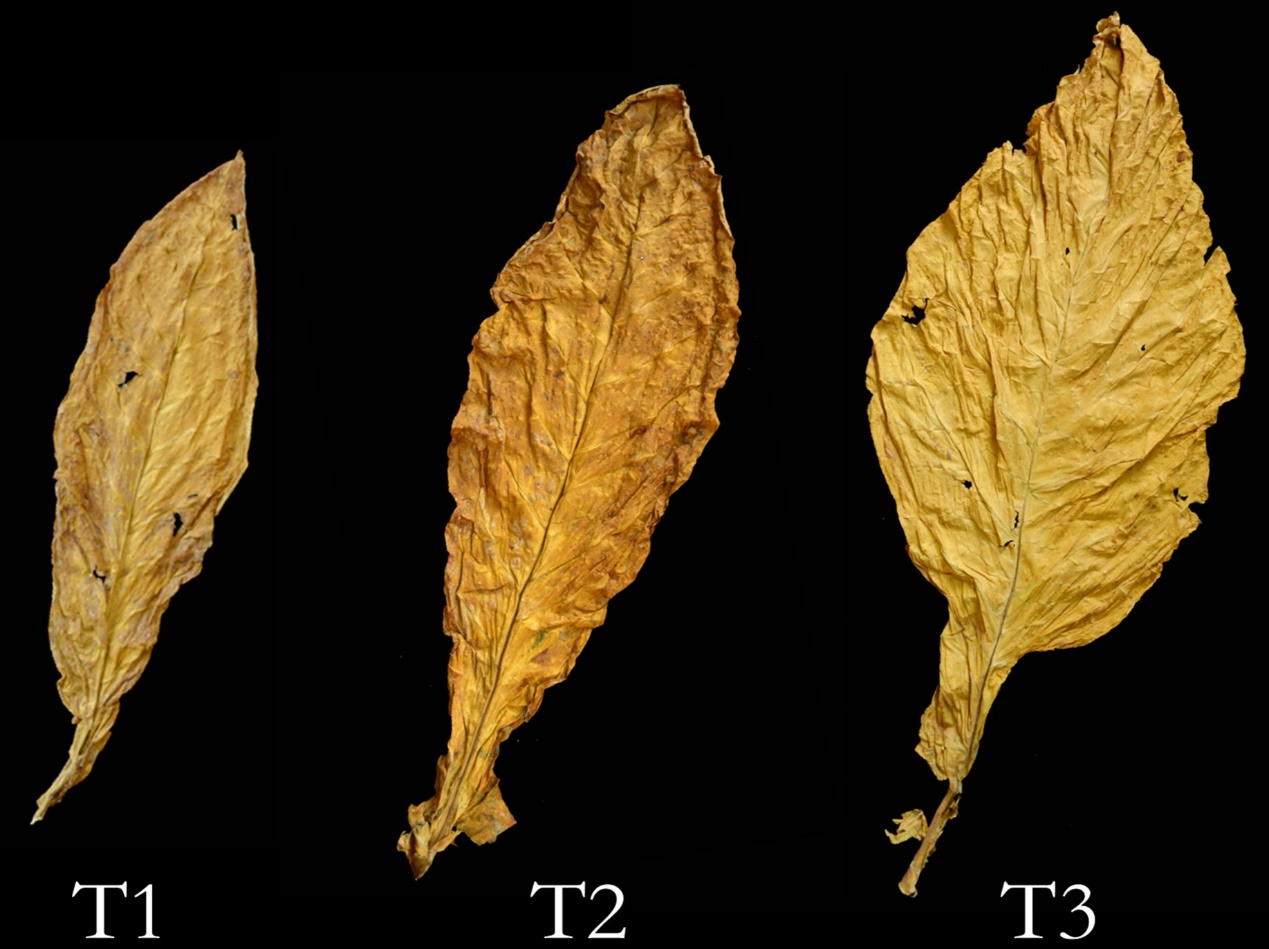


**FIGURE S1** Comparison of appearance of flue-cured tobacco after curing with different nitrogen fertilization rates. T1, N 18.9 kg·ha^-1^ + Rot farm manure 15000 kg·ha^-1^; T2, N 27 kg·ha^-1^ + Rot farm manure 15000 kg·ha^-1^, which is the normal local nitrogen fertilization rates; T3, N 35.1 kg·ha^-1^ + Rot farm manure 15000 kg·ha^-1^.


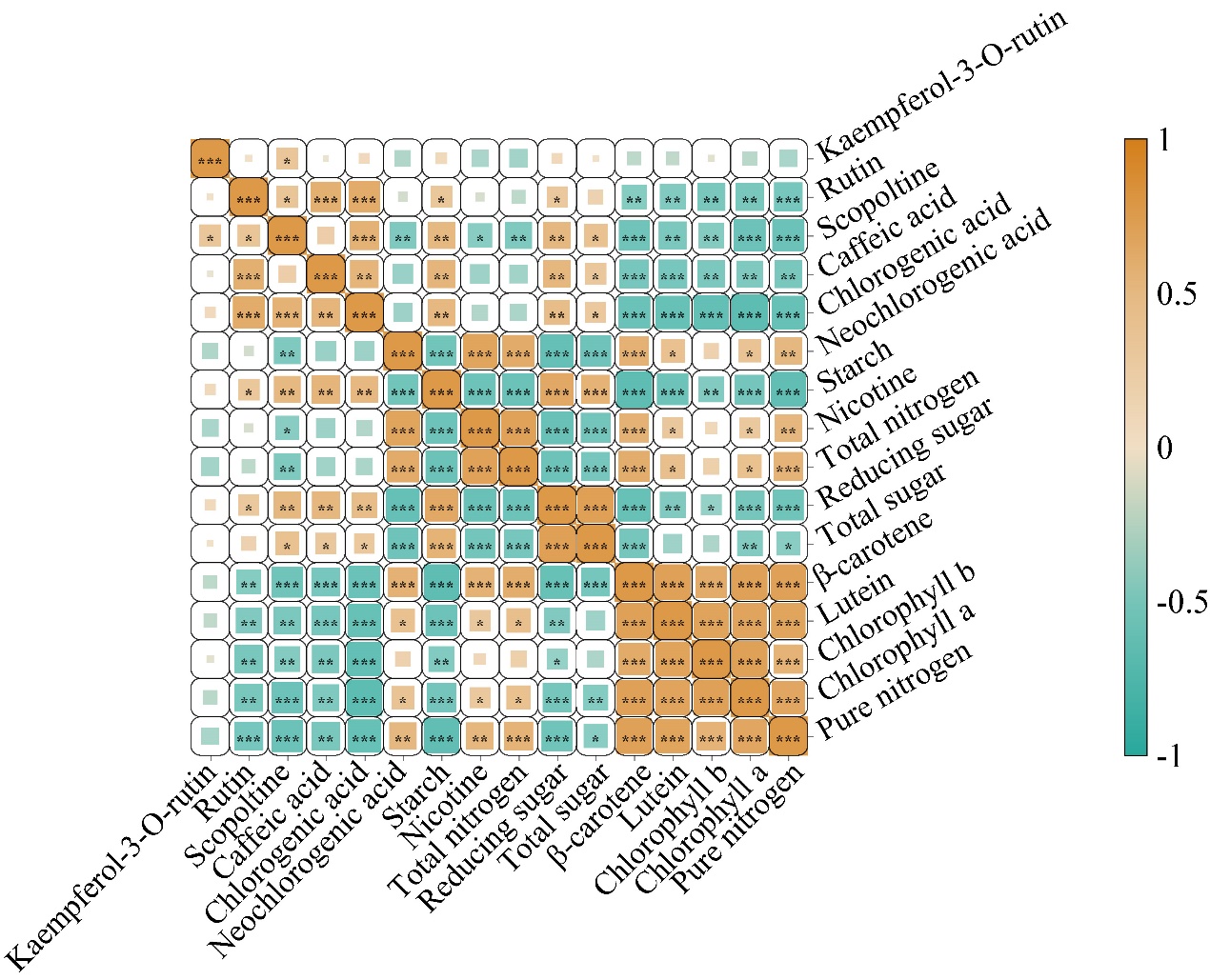


**FIGURE S2** Correlation analysis of factors which control economic traits of flue-cured tobacco leaves.
